# A generic whole body physiologically based pharmacokinetic model for therapeutic proteins in PK-Sim

**DOI:** 10.1007/s10928-017-9559-4

**Published:** 2017-12-12

**Authors:** Christoph Niederalt, Lars Kuepfer, Juri Solodenko, Thomas Eissing, Hans-Ulrich Siegmund, Michael Block, Stefan Willmann, Jörg Lippert

**Affiliations:** 1 Clinical Pharmacometrics, Bayer AG Pharmaceuticals, 51368 Leverkusen, Germany; 2 Clinical Pharmacometrics, Bayer AG Pharmaceuticals, 42113 Wuppertal, Germany

**Keywords:** Physiologically based pharmacokinetic modelling, PBPK, Therapeutic proteins, Antibodies, Biologics

## Abstract

Proteins are an increasingly important class of drugs used as therapeutic as well as diagnostic agents. A generic physiologically based pharmacokinetic (PBPK) model was developed in order to represent at whole body level the fundamental mechanisms driving the distribution and clearance of large molecules like therapeutic proteins. The model was built as an extension of the PK-Sim model for small molecules incorporating (i) the two-pore formalism for drug extravasation from blood plasma to interstitial space, (ii) lymph flow, (iii) endosomal clearance and (iv) protection from endosomal clearance by neonatal Fc receptor (FcRn) mediated recycling as especially relevant for antibodies. For model development and evaluation, PK data was used for compounds with a wide range of solute radii. The model supports the integration of knowledge gained during all development phases of therapeutic proteins, enables translation from pre-clinical species to human and allows predictions of tissue concentration profiles which are of relevance for the analysis of on-target pharmacodynamic effects as well as off-target toxicity. The current implementation of the model replaces the generic protein PBPK model available in PK-Sim since version 4.2 and becomes part of the Open Systems Pharmacology Suite.

**Electronic supplementary material:**

The online version of this article (10.1007/s10928-017-9559-4) contains supplementary material, which is available to authorized users.

## Introduction

Whole body physiologically based pharmacokinetic (PBPK) models contain an explicit representation of those organs and tissues that have relevant impact on absorption, distribution, metabolism and elimination (ADME) of a drug [1–7]. The parametrization of PBPK models represents physiological and anatomical information about the organism as well as substance-specific properties of the drug. Physiological data used are, for example, blood flow rates and the volumes of cellular, interstitial and vascular spaces of the relevant organs. The drug-specific parameterization is based on physicochemical properties and in vitro or in vivo experiments that provide various information, e.g., on distribution, metabolism, or clearance [3, 4, 6, 7]. PBPK models are used during pre-clinical and clinical drug development for mechanistic analysis of drug ADME processes, for cross-species extrapolation or for scaling to special populations (e.g., patients with a specific disease states or children) [1–6, 8].

Therapeutic proteins are an increasingly important class of drugs [9–11]. For example, monoclonal antibodies are used for different indications including cancer, inflammatory and autoimmune diseases [11]. More than 20 monoclonal antibodies have been approved in the US from 2014 to 2016 and more than 50 monoclonal antibodies are currently (early 2017) undergoing late stage clinical investigation [12]. Furthermore, engineered antibody fragments with tailored pharmacokinetic properties and functionality gain interest as diagnostic and therapeutic agents [9].

Compared to small molecule drugs, there are characteristic differences in the pharmacokinetics of therapeutic proteins mainly due to their large molecular size [13–16]. PBPK models must therefore take into account the special mechanisms that govern the pharmacokinetics of protein therapeutics, mechanisms that can often be neglected for small molecules. For example, the exchange of drug across the vascular endothelium and the return of drug by the lymph flow from the interstitial space of the organs to the systemic circulation are relevant processes for therapeutic proteins. These two processes influence the volume of distribution for proteins, and are generally considered in published PBPK models of therapeutic proteins [17–31]. Due to the, in comparison, rapid diffusion of small compounds across the vascular walls and within tissues, these processes are not relevant for a typical small molecule drug. Another relevant process for therapeutic proteins is the catabolism within endosomal space and the protection from catabolism by the neonatal Fc receptor (FcRn), relevant for antibodies or albumin fusion proteins. Hence this too needs to be considered for PBPK models of therapeutic proteins [19–21, 23–25, 27, 30–32].

The aim of the current work is to extend the established PBPK model in PK-Sim [33–36] which was designed for small molecule drugs, to allow simulation of macromolecules such as protein therapeutics in one comprehensive pharmacokinetic modeling framework. The current implementation of the model replaces the unpublished generic protein PBPK model available in PK-Sim since version 4.2 providing an updated parameterization using new experimental data [29] and an explicit representation of drug–FcRn binding. The model becomes part of the open source Open Systems Pharmacology Suite (www.open-systems-pharmacology.org).

Based on the generic model for small molecules, the generic model for proteins contains extensions to represent generally relevant processes as the passive exchange across the vascular endothelium, the return of a drug by the lymph flow to the systemic circulation as well as the active catabolism within endosomal space and the protection from catabolism by FcRn which is relevant for an important class of proteins. Any other active processes relevant for a specific drug can be added using the Open Systems Pharmacology Suite [37]. Examples of such processes include target-mediated disposition and clearance [21, 30, 31, 38, 39] and immunogenicity [40, 41].

## Methods

### PBPK model structure

#### General model description

The PBPK model for proteins was built as an extension of the PBPK model for small molecule drugs implemented within the software PK-Sim [33–36] (http://open-systems-pharmacology.org). As for the PBPK model for small molecules, it contains 15 organs or tissues and distinct blood pool compartments. Specifically, the represented organs/tissues include adipose tissue, brain, bone, gonads, heart, kidneys, large intestine, small intestine, liver, lung, muscle, pancreas, skin, spleen, stomach as well as the blood pool compartments arterial blood, venous blood and portal vein blood. For the substructure of the small and large intestine representation refer to [36]. Each organ consists of sub-compartments representing the plasma, blood cells (which together form the vascular space), interstitial space and cellular space. All physiologic parameters (organ volumes, fraction of interstitial, vascular and cellular space of the organs, blood flow rates and hematocrit) for the different species were used from the small molecule model without changes [42–44].

For the PBPK model for proteins, an additional compartment was added for each organ representing the endosomes and lysosomes within vascular endothelial cells. In this endosomal space compartment, lysosomal degradation and high affinity FcRn binding is located. Since the model was derived from a PBPK model for small molecule drugs, cellular space is explicitly represented. However, the permeability for passive diffusion into cells was neglected for all drugs in the present study, since this process is not relevant for macromolecules or very hydrophilic drugs like inulin. The explicit representation of cellular space is relevant to describe active uptake into cells when necessary (e.g., internalization of protein drug bound to membrane surface receptors). Additionally, organ-specific lymph flow (*L*_*org*_) was integrated into the model for protein therapeutics connecting the interstitial space of each organ to the venous blood pool using the rate equation

1 $$J_{ip,org} = L_{org} \cdot C_{i,org}$$

with *J*_*ip*,*org*_ being the flux rate of drug from the interstitial space of organ *org* to the central venous blood plasma pool and *C*_*i*,*org*_ the drug concentration in interstitial space.

**Fig. 1:**
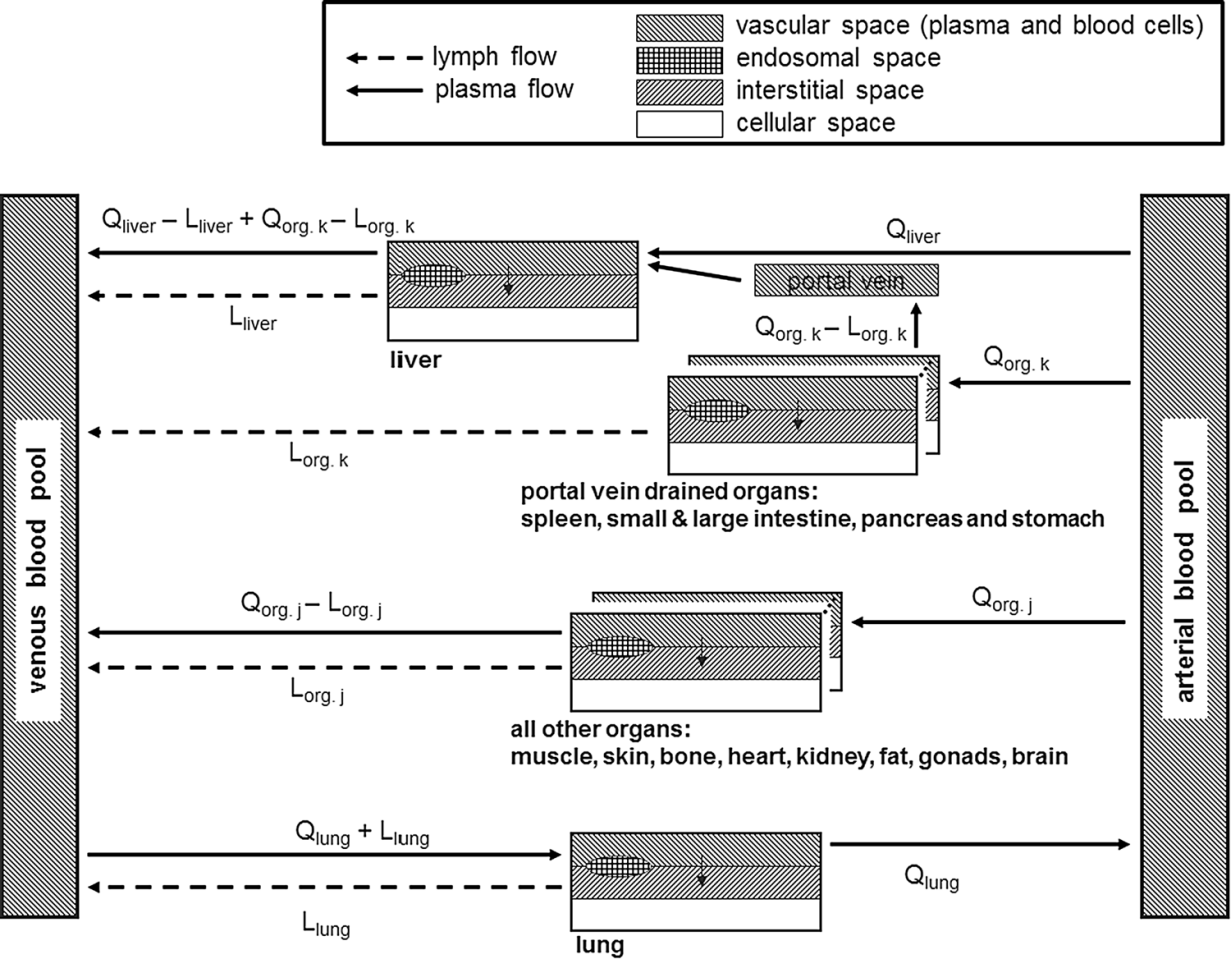
Scheme of the PBPK model for protein therapeutics showing connection of organs by blood and lymph flow. For the substructure of the small and large intestine cf. [36]

A scheme of the PBPK model structure for protein therapeutics showing how organs are connected by blood and lymph flow is given in Fig. 1.

#### Extravasation by the two-pore model

To describe the transcapillary exchange of the drug between plasma and interstitial space in each organ, the two-pore formalism [45, 46] was applied. According to this theory, the barrier between plasma and interstitial space is described as a membrane consisting of two types of pores: large and small ones. Macromolecules can pass through these pores by convection as well as diffusion.

The exchange of macromolecules (amount per time) between plasma and interstitial space of each organ by the two-pore formalism is given by the following equation:

2 $$\begin{aligned} J_{vi,org} & = f_{u} \left( {J_{L,org} \cdot \left( {1 - \sigma_{L,org} } \right) \cdot C_{v,org}\,+\,PS_{L,org} \cdot \left( {C_{v,org} - \frac{{C_{i,org} }}{{K_{iv,org} }}} \right) \cdot \frac{{Pe{\kern 1pt}_{L,org} }}{{e^{{Pe{\kern 1pt}_{L,org} }} - 1}}} \right. \\ & \quad \left. {\,+\,\,J_{S,org} \cdot \left( {1 - \sigma_{S,org} } \right) \cdot C_{v,org}\,+\,PS_{S,org} \cdot \left( {C_{v,org} - \frac{{C_{i,org} }}{{K_{iv,org} }}} \right) \cdot \frac{{Pe_{S,org} }}{{e^{{Pe_{S,org} }} - 1}}} \right) \\ \end{aligned}$$

with *J*_*vi*,*org*_: flux rate (amount per time) of drug from plasma to interstitial space in organ *org*, *f*_*u*_: fraction unbound in plasma, *C*_*v*,*org*_: concentration of drug in plasma of organ *org*, *C*_*i*,*org*_: concentration of compound in interstitial space of organ *org*, *J*_*L*,*org*_, *J*_*S*,*org*_: transcapillary fluid flow rate via large/small pores for organ *org*, *γ*_*L*,*org*_, *γ*_*S*,*org*_: reflection coefficients for large/small pores for organ *org*, *Pe*_*L*,*org*_, *Pe*_*S*,*org*_: Peclet number for large/small pores in organ *org*, *PS*_*L*,*org*_, *PS*_*S*,*org*_: product of permeability and surface area for large/small pores for organ *org*, *K*_*iv*,*org*_: partition coefficient between interstitial space and plasma for organ *org*.

The fraction unbound in plasma (*f*_*u*_) was set to 1 for all simulations. The factor was included in order to allow simultaneous description of small molecules in the same framework.

According the two-pore formalism, the transcapillary fluid flow rate for large and small pores is calculated by

3 $$J_{L,org} = J_{iso,org} + \alpha_{L,org} L_{org} ,$$

4 $$J_{S,org} = - J_{iso,org} + \alpha_{S,org} L_{org}$$

respectively, where *L*_*org*_ is the lymph flow and *α*_*L*,*org*_ and *α*_*S*,*org*_ are the fractions of flow via large and small pores, respectively, in organ *org*. The fluid recirculation flow rate *J*_*iso*,*org*_ describes the flow under isogravimetric conditions, i.e., without net fluid flow across the vascular wall.

The reflection coefficients for large and small pores depend on the drug solute radius and were calculated by the equations given in [46]:

5 $$\sigma_{L,org} = 1 - \frac{{(1 - \gamma_{L,org} )^{2} \cdot [2 - (1 - \gamma_{L,org} )^{2} ] \cdot (1 - \gamma_{L,org} /3)}}{{1 - 1/3 \cdot \gamma_{L,org} + 2/3 \cdot \gamma_{L,org}^{2} }},$$

6 $$\sigma_{S,org} = 1 - \frac{{(1 - \gamma_{S,org} )^{2} \cdot [2 - (1 - \gamma_{S,org} )^{2} ] \cdot (1 - \gamma_{S,org} /3)}}{{1 - 1/3 \cdot \gamma_{S,org} + 2/3 \cdot \gamma_{S,org}^{2} }}$$

whereas *γ*_*L*/*S*,*org*_ is the ratio of solute radius (a_e_) and endothelial pore radius for large (*r*_*L*,*org*_) and small pores (*r*_*S*,*org*_), respectively: *γ*_*L*,*org*_ = *a*_*e*_/*r*_*L*,*org*_ and *γ*_*S*,*org*_ = *a*_*e*_/*r*_*S*,*org*_.

The Peclet numbers, describing the ratio of convective and diffusive transport, are given by the equations [46]

7 $$Pe_{L,org} = J_{L,org} \cdot \frac{{1 - \sigma_{L,org} }}{{PS_{L,org} }},$$

8 $$Pe_{S,org} = J_{S,org} \cdot \frac{{1 - \sigma_{S,org} }}{{PS_{S,org} }}.$$

The rate of diffusion depends on the permeability–surface area products for small and large pores in Eq. (2), *PS*_*S*,*org*_, and *PS*_*L*,*org*_, respectively. The compound dependent permeabilities (*P*_*S*,*org*_, and *P*_*L*,*org*_) and the endothelial surface areas (*S*_*org*_) are calculated separately as described in the following section. Since the available literature for capillary surface areas for the different organs and species is rather limited, the following heuristic is used to calculate the capillary surface area for the different organs:

9 $$S_{org} = k \cdot f_{vas,org} \cdot V_{org}$$

with *k* being a constant of proportionality, *f*_*vas*,*org*_ being the fraction of vascular space of an organ, and *V*_*org*_ being the volume of an organ. The idea behind this heuristic is the following: with the assumption that the morphology of the vascular tree is similar in each organ, the specific surface area per organ volume can be estimated by the capillary density of an organ, which in turn can be estimated by the fraction of the vascular space of an organ. The constant of proportionality k = 950,000 cm^2^/l was adjusted to the estimated total capillary surface area of the vascular endothelium for humans (300 m^2^ [47]).

The permeabilities for small and large pores for each organ (*P*_*S*,*org*_, and *P*_*L*,*org*_, respectively) are calculated in the following way [46, 48]:

10 $$P_{S,org} = \xi_{S,org} \cdot \frac{D}{L}\frac{{A_{S,org} }}{{S_{org} }},\quad P_{L,org} = \xi_{L,org} \cdot \frac{D}{L}\frac{{A_{L,org} }}{{S_{org} }},$$

where D is the free diffusion coefficient of the solute, *ξ*_*S*,*org*_ and *ξ*_*L*,*org*_ are the ratios of the effective pore areas available for restricted diffusion through circular holes and the total cross sectional pore areas for small and large pores, respectively, *A*_*S*,*org*_ and *A*_*L*,*org*_ are the total cross sectional pore areas for small and large pores for the different organs, respectively, L is the effective thickness of the endothelial membrane and *S*_*org*_ are the capillary surface areas of the different organs. A comparison of values for the capillary surface area and the permeability–surface area product calculated by these equations for different organs to experimental values can be found in Tables S2 and S3 of the supplemental material, respectively.

The diffusion coefficient of the solute is calculated by the Stokes–Einstein relation

11 $$D = \frac{RT}{{6\pi \cdot N_{a} \cdot a_{e} \cdot \eta }},$$

where RT = 2.58E5 N cm/mol is the gas constant–body temperature (37 °C) product, N_a_ = 6.022E23/mol is the Avogadro constant, η = 1.17E−9 N min/cm^2^ is the viscosity of water, and a_e_ is the solute radius.

The dimensionless parameter ξ_S_ and ξ_L_ are calculated as

12 $$\xi_{S,org} = \frac{{(1 - \gamma_{S,org} )^{9/2} }}{{1 - 0.3956 \cdot \gamma_{S,org} + 1.0616 \cdot \gamma_{S,org}^{2} }}\quad {\text{and}}\quad \xi_{L,org} = \frac{{(1 - \gamma_{L,org} )^{9/2} }}{{1 - 0.3956 \cdot \gamma_{L,org} + 1.0616 \cdot \gamma_{L,org}^{2} }},$$

where *γ*_*S*,*org*_ = *a*_*e*_/*r*_*S*,*org*_ and *γ*_*L*,*org*_ = *a*_*e*_/*r*_*L*,*org*_ are the ratios of solute radius and pore radii of small and large pores, respectively.

The remaining factors are calculated via the hydraulic conductivity Lp_org_ of the endothelium in the different organs applying Poiseuille’s law:

13 $$\frac{{A_{L,org} }}{{L \cdot S_{org} }} = \alpha_{L,org} \cdot \frac{{8 \cdot \eta \cdot Lp_{org} }}{{r_{L,org}^{2} }},\quad {\text{and}}\quad \frac{{A_{S,org} }}{{L \cdot S_{org} }} = \left( {1 - \alpha_{L,org} } \right) \cdot \frac{{8 \cdot \eta \cdot Lp_{org} }}{{r_{S,org}^{2} }},$$

where *α*_*L*,*org*_ is the fraction of flow via large pores, η = 1.17E−9 N min/cm^2^ is the viscosity of water and *r*_*S*,*org*_ and *r*_*L*,*org*_ are the radii of small and large pores, respectively.

#### FcRn binding model

**Fig. 2:**
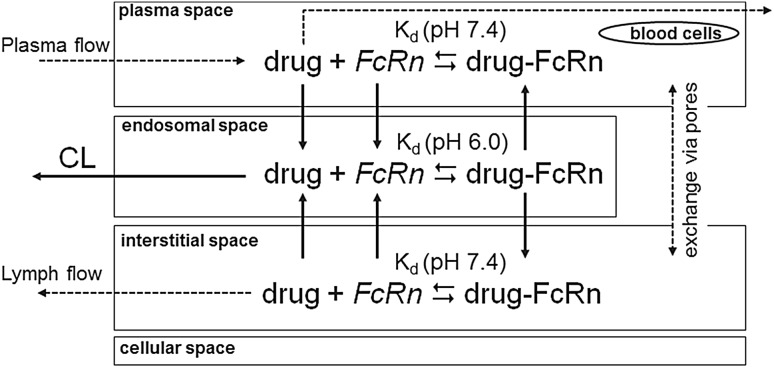
Representation of catabolism and protection from catabolism by binding to the FcRn receptor in each organ. Note that with the parameterization used in the present model, no uptake of drug from interstitial space and no recycling of drug–FcRn to interstitial space occur. For FcRn an effective pooled concentration within a simplified sub-model is considered (cf. text and Fig. 3). The exchange via pores is effective only for drug

The FcRn binding model is used to represent the catabolic clearance of a protein drug within the endosomal space and the protection from catabolism by FcRn binding which is relevant for antibodies and Fc or albumin fusion proteins. The schema of the FcRn binding model which is implemented in each organ is given in Fig. 2.

The representation of each organ within PK-Sim is extended by an additional compartment, the endosomal space. The endosomal space represents the region within the cells of the endothelial capillary walls where catabolism and high affinity binding to the FcRn receptor occurs (acidic environment). The volume of the endosomal space in each organ is calculated by the equation

14 $$V_{endo, org} = f_{endo} d_{e} S_{org} ,$$

where *V*_*endo*,*org*_ is the endosomal volume in each organ, *f*_*endo*_ is the fraction of endosomal space in the vascular endothelium, *S*_*org*_ is the vascular surface area and *d*_*e*_ is the thickness of vascular endothelium (cf. “Physiological parameters” section for values used).

FcRn binding is explicitly represented, i.e., the drug can reversibly bind to FcRn forming the drug–FcRn complex. The drug–FcRn-complex is recycled to plasma and interstitial space, while drug which is not bound to FcRn is subject to the endosomal clearance. In the neutral environment of plasma and interstitial space the binding to FcRn is characterized with the low affinity dissociation constant for neutral environment (in the standard version of the model effectively set to infinity) and the drug–FcRn-complex dissociates.

**Fig. 3:**
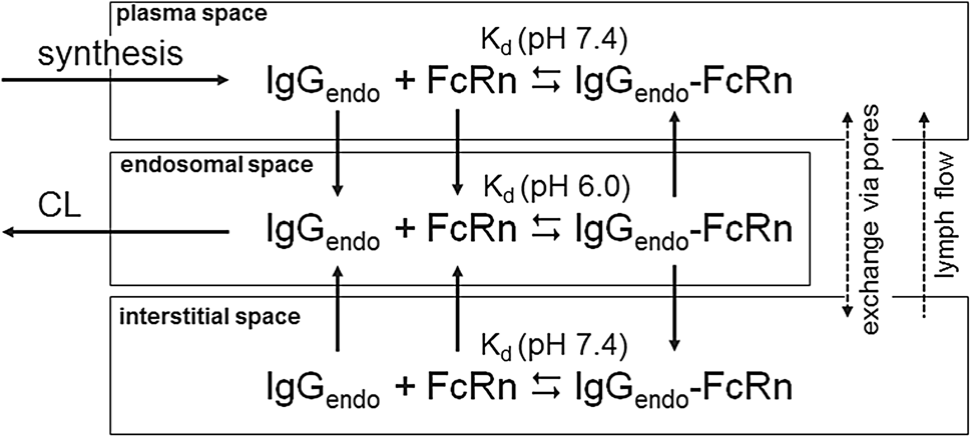
Representation of the sub-model structure for the endogenous IgG and FcRn. Note that with the parameterization used in the present model, no uptake of endogenous IgG from interstitial space and no recycling of endogenous IgG–FcRn to interstitial space occur. The exchange via pores and lymph flow is effective only for endogenous IgG

Endogenous IgG is also represented in the model, competing with the drug for the FcRn receptor. In order to allow the algebraic calculation of the interstitial and endosomal concentration of the endogenous IgG for the physiological steady state without drug (i.e., of the initial concentrations at time 0), the endogenous IgG together with the FcRn receptor is represented within a simplified sub-model structure (cf. Fig. 3). Based on a quasi steady-state approximation, this sub-model lumps the plasma, the interstitial and endosomal space of the whole organism each into one effective compartment. That is, for the endogenous IgG no differentiation of the single tissues is taken into account and the drug within the tissue compartments reacts with a pooled concentration of FcRn. As for the drug, the fraction of endogenous IgG which is not bound to FcRn is catabolized within the endosomal space. To compensate for this clearance of endogenous IgG, a zero order synthesis continuously releases endogenous IgG to the plasma compartment of the endogenous IgG representation. The equations for the steady state concentration of endogenous IgG, FcRn and IgG_endo_–FcRn complex without drug are used as initial conditions and are given in the supplemental material, Sect. 5. This simplified sub-model structure avoids simulation of the PBPK model without drug to determine the initial endogenous IgG concentrations numerically before simulating the drug.

Since this sub-model represents all organs, it has the same structure as a standard organ (including vascular exchange via the two-pore formalism) and the corresponding parameters (volumes of the plasma compartment, interstitial space and endosomal space as well as the lymph and recirculation flow rates and the vascular surface area) are just calculated as the sum over all organs of the respective parameters.

The mass transfer of the drug from plasma and interstitial space to the endosomal space is described in each organ by the following two equations. The mass transfer of the endogenous IgG in the sub-model is also described by the same equations:

15 $$\frac{{dn^{comp} }}{dt} = f_{vas}^{up} \cdot k_{up} \cdot V_{end} \cdot C_{pls}^{comp} ,$$

16 $$\frac{{dn^{comp} }}{dt} = \left( {1 - f_{vas}^{up} } \right) \cdot k_{up} \cdot V_{end} \cdot C_{\text{int}}^{comp} ,$$

where *n* ^*comp*^ is the amount of substance of the drug or of the endogenous IgG, $f_{vas}^{up}$ is the fraction of endosomal uptake from plasma, *k*_*up*_ is the endosomal uptake rate constant, *V*_*end*_ is the endosomal volume, $C_{pls}^{comp}$ is the drug or endogenous IgG concentration in plasma, and $C_{\text{int}}^{comp}$ is the drug or endogenous IgG concentration in the interstitial space. Note that with the parameterization described in “Physiological parameters” section, effectively no uptake of drug or endogenous IgG from the interstitial space occurs.

The recycling of the FcRn complex from the endosomal space back to plasma and interstitial space is described by the following equations:

17 $$\frac{{dn^{comp {-} FcRn} }}{dt} = f_{vas}^{rec} \cdot k_{rec} \cdot V_{end} \cdot C_{end}^{comp {-} FcRn} ,$$

18 $$\frac{{dn^{comp {-} FcRn} }}{dt} = \left( {1 - f_{vas}^{rec} } \right) \cdot k_{rec} \cdot V_{end} \cdot C_{end}^{comp {-} FcRn} ,$$

where *n* ^*comp–FcRn*^ is the amount of substance of the drug–FcRn or endogenous IgG–FcRn complex, $f_{vas}^{rec}$ is the fraction of recycling of the FcRn complex from endosomal space to plasma, *k*_*rec*_ is the recycling rate constant, and $C_{end}^{comp {-} FcRn}$ is the concentration of the drug–FcRn or endogenous IgG–FcRn complex in the endosomal space. Note that with the parameterization described in “Physiological parameters” section, effectively no recycling of drug–FcRn or endogenous IgG–FcRn to interstitial space occurs.

The specific clearance of the drug not bound to FcRn from the endosomal space is calculated as the difference of the uptake and recycling rate constants *k*_*up*_ − *k*_*rec*_, thus clearance from the endosomal space is given by the equation:

19 $$\frac{{dn^{drug} }}{dt} = \left( {k_{up} - k_{rec} } \right) \cdot V_{end} \cdot C_{end}^{drug} .$$

For the endogenous IgG sub-model the rate equations are analogously to those given above for the drug. The parameters $f_{vas}^{up} ,$ *k*_*up*_ and *k*_*rec*_ are assumed to be the same for all organs.

The FcRn binding reaction for the drug and the endogenous IgG in plasma, interstitial, or endosomal space is described by the equation:

20 $$\frac{{dC^{comp {-} FcRn} }}{dt} = - \frac{{dC^{comp} }}{dt} = - \frac{{dC^{FcRn} }}{dt} = k_{ass} \cdot C^{comp} \cdot C^{FcRn} - K_{d} \cdot k_{ass} \cdot C^{comp {-} FcRn}$$

whereby *C* ^*comp*^ is the concentration of the drug in the different organs or of endogenous IgG in the sub-model for the endogenous IgG/FcRn, *C* ^*FcRn*^ is the concentration of FcRn in the sub-model for the endogenous IgG/FcRn, *C* ^*comp*–*FcRn*^ is the concentration of the FcRn complex of drug in the different organs or endogenous IgG in the sub-model for the endogenous IgG/FcRn, *k*_*ass*_ is the association rate constant for FcRn binding and *K*_*d*_ is the dissociation constant for FcRn binding.

### Model parameters

#### Physiological parameters

The original database for anatomical and physiological parameters in PK-Sim was updated with the parameters specific for the extended model for protein therapeutics. Values of physiological and biochemical parameters were taken from literature or derived from literature data. The parameters which describe the vascular endothelium and which are used to calculate the reflection coefficients σ_L_ and σ_S_ as well as the permeabilities P_L_ and P_S_ are given in Table 1. The pore radii and fractions of flow via large pores used in the present model represent two different types of vascular endothelium: one is the continuous (non-fenestrated or fenestrated), the other the discontinuous endothelium [49–51].

**Table 1:** Parameters used describing vascular endothelium in different organs

Organs	Hydraulic conductivity, Lp (ml/min/N)	Fraction of flow via large pores, α_L_	Radius of small pores, r_S_ (nm)	Radius of large pores, r_L_ (nm)
Bone	3.24E−04^a^	0.05^h^	4.5^h^	25^h^
Brain	1.80E−06^b^	0.05	4.5	25
Fat	3.24E−04^a^	0.05	4.5	25
Gonads	3.24E−04^a^	0.05	4.5	25
Heart	5.16E−04^b^	0.05	4.5	25
Kidney	4.5E−03^d^	0.05	4.5	25
Large intestine	6.73E−03^e^	0.05	4.5	25
Liver	1.40E−03^c^	0.80^i^	9^i^	33^i^
Lung	2.04E−04^b^	0.05	4.5	25
Muscle	3.24E−04^f^	0.05	4.5	25
Pancreas	1.16E−03^e^	0.05	4.5	25
Skin	7.01E−04^f^	0.05	4.5	25
Small intestine	5.54E−03^e^	0.05	4.5	25
Spleen	1.40E−03^c^	0.80^i^	9^i^	33^i^
Stomach	1.43E−03^e^	0.05	4.5	25
Sub-model for endogenous IgG	6.65E−04^g^	0.05	4.5	25

^a^No literature data available, same value as for muscle was used

^b^Values from [88]

^c^Lp for discontinuous endothelium was calculated from the capillary filtration coefficient for liver measured by Granger et al. [89] and the endothelial surface area of the respective organ calculated with the described model heuristic

^d^Value for peritubular capillaries from [90]

^e^Calculated from the capillary filtration coefficient of the respective organ measured by Granger et al. [89] and the endothelial surface area calculated with the described model heuristic

^f^Calculated from the capillary filtration coefficient of the respective organ measured by Renkin et al. [91] and the endothelial surface area calculated with the described model heuristic

^g^Value calculated as vascular surface area weighted mean over all tissues

^h^Values for continuous endothelium taken from [46]

^i^Values for discontinuous endothelium taken from liver data of [92]

For all organs, the partition coefficients *K*_*iv*,*org*_ are calculated using the equation for the interstitial/plasma partition coefficient implemented in PK-Sim [52]. Assuming a fraction unbound in plasma of 1 for all compounds, this equation yields a value of approximately 1 for all species and organs (*K*_*iv*,*org*_ = 0.96). The value slightly smaller than 1 indicates, that the effective volume fraction accessible for distribution is slightly smaller in the interstitial space than in plasma due components into which the drug does not partition into (neither water nor protein) [52].

The parameters characterizing the vascular endothelium given in Table 1 (pore radii, fraction of flow via large pores, hydraulic conductivity) are assumed to be species independent, i.e., the same values are used for all animal species and humans.

To facilitate the use of physiologically reasonable lymph flow rates for all animal species and humans, the lymph flow *L*_*org*_ of each organ was expressed as fraction of plasma flow:

21 $$L_{org} = f_{lymph,org} \cdot Q_{blood,org} \cdot (1 - HCT)$$

with *Q*_*blood*,*org*_ being the blood flow and *HCT* being the hematocrit.

Similarly, the recirculation flow *J*_*iso*,*org*_ was expressed as a fraction of lymph flow via small pores. Interestingly, during model development we found that the plasma PK for larger species than mice (especially for humans) was better described when assuming a reduced fraction of lymph flow. Thus an additional empirical organ volume based allometric scaling factor $(V_{org}^{species} /V_{org}^{mouse} )^{{(\gamma_{Jiso} - 1)}}$ was used to calculate the recirculation flow rate *J*_*iso*,*org*_, using the scaling exponent *γ*_*Jiso*_, such as:

22 $$J_{iso,org} = f_{Jiso,org} \cdot \left( {1 - \alpha_{L,org} } \right) \cdot L_{org} \cdot \left( {V_{org}^{species} /V_{org}^{mouse} } \right)^{{(\gamma_{Jiso} - 1)}} .$$

The parameters *f*_*lymph*,*org*_, *f*_*Jiso*,*org*_ and *γ*_*Jiso*_ were fitted to experimental tissue concentration–time profiles (see “Parameter estimation” section). Since pore sizes and densities differ among organs, *f*_*lymph*,*org*_ and *f*_*Jiso*,*org*_ were allowed to be different for different organs.

The lymph and recirculation flow rates of the sub-compartments of the small and large intestine (mucosal segments) [36] were calculated from the total lymph and recirculation flow of the small and large intestine, respectively, assuming that the flows of the segments are proportional to the volume fraction of the segment (V_segment_/V_small intestine_, V_segment_/V_large intestine_, respectively).

To calculate the volume of the endosomal space, the following parameters were used: the fraction of endosomal space in endothelium (*f*_*endo*_) was set to a value of 0.2 [27] and the thickness of endothelium was set to *d*_*e*_ = 0.3 µm [53].

Drug extravasation is represented by the two-pore formalism in the current PBPK model. Structurally, net extravasation can additionally occur via the endosomal space by drug uptake and FcRn mediated recycling from and to plasma and interstitial space. In order to prevent the net extravasation via the endosomal space and to restrict extravasation to the two-pore mechanism, $f_{vas}^{up}$ and $f_{vas}^{rec}$ were both set to 1 for all simulations in all species, i.e., no uptake from interstitial space to the endosomal space and no recycling to interstitial space was taken into account.

The only parameters which are explicitly species dependent are related to the FcRn binding model. The plasma concentrations of endogenous IgG and the affinities of endogenous IgG to FcRn are taken from literature (cf. Table 2). The endosomal concentrations of FcRn in mice, monkeys and humans were fitted to experimental PK data (see Table 7).

**Table 2:** Species dependent a priori parameters used within the FcRn binding model

Parameters	Mouse	Monkey	Human
Plasma concentration of endogenous IgG (µmol/l)	18^a^	75^b^	70^c^
K_d_ for binding of endogenous IgG to FcRn receptor in endosomal space (µmol/l)	0.75^d^	0.132^b^	0.63^d^

^a^Ref. [93]

^b^Ref. [94]

^c^Ref. [95]

^d^Ref. [96]

The K_d_ value for binding of endogenous IgG to FcRn in plasma and interstitial space was set to a very high value (99,999 µmol/l, representing virtually no binding) for all simulations since for wild type antibodies–FcRn binding in neutral environment is negligible [54].

The standard PK-Sim model does not include tumor tissue. In order to simulate drugs applied to xenograft mice, the generic PBPK model was extended by a tumor organ with the same structure as other organs in PK-Sim. The parameter values used for the tumor organ in the current study for the simulation of BAY 79-4620 are given in Table 3.

**Table 3:** A priori PBPK parameter used for the tumor tissue

Volume (ml)	0.2
Blood flow (ml/min/g)	0.21^a^
Fraction of vascular space	0.05^b^
Fraction of interstitial space	0.45^c^
Lp (ml/min/N)	1.6E−03^d^
α_L_	0.05^e^
r_S_ (nm)	4.5^e^
r_L_ (nm)	25^e^

^a^Ref. [18]

^b^Typical value from [97]

^c^Typical value from [98]

^d^Ref. [99]

^e^Standard value for continuous endothelium [46]

Additionally, the target of BAY 79-4620, carbonic anhydrase IX (CA IX), was represented in the interstitial space of the tumor organ in order to describe target mediated tumor disposition for BAY 79-4620. The turnover half-live of CA IX was set to 38 h [55] and the interstitial concentration (initial condition) of CA IX was set to a value of 0.26 µmol/l, which was estimated from the CA IX density of 2.4E5 per HT-29 cell [56]. The internalization of the complex BAY 79-4620–CA IX leading to target mediated elimination was represented by a first order process and the internalization rate constant was fitted to experimental data.

#### Drug specific parameters

The PBPK model was developed and evaluated with compounds of different size and affinity to FcRn: the antibodies 7E3, MEDI-524, MEDI-YTE, CDA1 and tefibazumab (each with a molecular weight of 150 kDa), the antibody–drug conjugate (ADC) BAY 79-4620 (152 kDa), a domain antibody (25.6 kDa) and inulin (5.5 kDa).

If no further (e.g., target binding) processes are involved, the hydrodynamic radius of the drug and the dissociation constant for binding to FcRn [K_d_(FcRn)] are the only drug specific input parameters used to define the extravasation and endosomal clearance together with the physiological parameters as described above.

The values for these parameters used for the compounds in the present study are given in Tables 4 and 5, respectively.

**Table 4:** Values for hydrodynamic compound radius used

Compounds	Hydrodynamic radius (nm)
7E3, BAY 79-4620, MEDI-524, MEDI-YTE, CDA1, Tefibazumab	5.34^a^
Domain antibody	2.43^b^
Inulin	1.39^c^

^a^Value for antibody from [92]

^b^Calculated based on molecular weight, see supplemental material, Sect. 1

^c^Ref. [100]

**Table 5:** Dissociation constants for FcRn binding in endosomal space for the compounds used in the present study

Compounds	Ab types	FcRn types	K_d_ (µM)
7E3	Mouse	Mouse	0.75^a^
BAY 79-4620	Humanized	Mouse	12.7^c^
MEDI-524	Humanized	Cynomolgus	1.196^b^
MEDI-524-YTE	Humanized, Fc variant	Cynomolgus	0.134^b^
CDA1 and Tefibazumab	Human, humanized	Human	0.63^a^
Domain antibody	No Fc region	–	999,999^d^
Inulin	Polysaccharide	–	999,999^d^

^a^Ref. [96]

^b^Ref. [59]

^c^Fitted to PK data

^d^Virtually no FcRn binding due to missing Fc region

As for endogenous IgG, the K_d_(FcRn) value for binding in neutral space was set to a high value (999,999 µM) resulting in virtually no FcRn binding for all compounds.

For the simulations of BAY 79-4620 a reversible binding reaction to its surface receptor target was added to the generic structure as an additional active process. For the affinity to the target the experimental value of K_d_ = 4 nM was used in the model [57].

Due to the relatively small size the domain antibody is subject to restricted renal filtration. Thus, an additional clearance process was added to the plasma compartment of the kidney for the domain antibody. The renal clearance was defined as *CL*_*ren*_ = *f*_*GFR*_·*GFR*, where GFR is the glomerular filtration rate (0.28 ml/min in mice [42]) and the glomerular filtration coefficient *f*_*GFR*_ was optimized by fitting to the experimental data.

For the inulin simulations, it was assumed that inulin is not catabolized in the endosomes. Thus the endosomal uptake (*k*_*up*_)—and in consequence the endosomal clearance—was set to zero for inulin. Also, the renal clearance was taken into account for inulin setting the glomerular filtration coefficient to 1 (GFR for rat 1.31 ml/min [42]).

### PK data used for parameter estimation

Plasma and tissue concentration versus time profiles were used to identify unknown parameters.

The following data sets were used.

#### Antibody–drug conjugate BAY 79-4620 in mice

BAY 79-4620 is an ADC consisting of a human IgG1 mAb directed against CA IX conjugated to monomethylauristatin E via a cathepsin cleavable vc linker [57]. Tissue distributions from an in-house quantitative whole body autoradiography study as well as from an in-house wet-tissue dissection study were used. For the autoradiography study, female nude mice (NMRI nu/nu), bearing HT-29 human colon carcinoma xenografts, were dosed intravenously with 1.25 mg/kg body weight of ^125^I-labeled BAY 79-4620. The distribution of total radioactivity in organs and tissues was determined by quantitative whole body autoradiography after sacrificing the mice (two per time) at various time points after administration. For the wet-tissue dissection study, female nude mice (NMRI nu/nu), bearing HT-29 human colon carcinoma xenografts, were dosed intravenously with 2 µCi (approx. 500 ng) of ^125^I-labeled BAY 79-4620. The distribution of total radioactivity in organs and tissues was determined after sacrificing the mice (three per time) at various time points after administration and dissection of the organs by determination of radioactivity using a gamma-counter.

#### Antibody 7E3 in wild-type and FcRn knockout mice

The murine monoclonal IgG1 antibody 7E3 has a high affinity for human platelet glycoprotein IIb/IIIa. However, it does not bind to the respective mouse glycoprotein [27]. The experimental plasma and tissue concentrations after single 8 mg/kg IV bolus injection of 7E3 were taken from a study by Garg and Balthasar [27]. Tissue concentrations of ^125^I-labeled 7E3 were determined from blotted dried tissues after sacrificing 3 mice per time point. Brain concentrations of the same antibody which were corrected for residual blood were taken from [58].

#### Domain antibody dAb_2_ in mice

In order to inform the model with data from a protein with a smaller solute radius, the plasma and tissue concentration–time profiles of a domain antibody dAb_2_ from [29] were used. The domain antibody dAb_2_ is a 25.6 kDa protein with no known binding to an endogenous target. The domain antibody was administered intravenously with a dose of 10 mg/kg and tissue concentrations were analyzed using quantitative whole body autoradiography. The kidney concentrations reported in [29] are not used during parameter estimation, since the kidney model structure of the present PBPK model does not represent tubular fluid. Since the domain antibody is cleared renally and the resulting contribution from the tubular fluid is not taken into account in the present model, the total kidney tissue concentrations cannot be expected to be adequately described.

#### MEDI-524 and MEDI-524-YTE in cynomolgus monkeys

MEDI-524 is a humanized anti-respiratory sincytial virus monoclonal antibody. MEDI-524-YTE is an Fc variant of this antibody with an approximately 10-fold increased affinity to cynomolgus FcRn at pH 6. The plasma concentration profiles of MEDI-524 and MEDI-524-YTE after a single intravenous (i.v.) dose of MEDI-524 or MEDI-524-YTE at 30 mg/kg were taken from [59].

#### CDA1 in human

CDA1 is a human monoclonal antibody (IgG1) against the toxin A of *Clostridium difficile.* The plasma concentration–time profiles after i.v. infusion of 5, 10 and 20 mg/kg CDA1 in healthy adults were taken from [60]. The data for the dosages 0.3 and 1 mg/kg were not used since the PK data could not be read with sufficient accuracy from the published figure.

### Parameter estimation

Parameters were optimized by fitting simultaneously to all plasma and tissue concentration–time profiles described above. Experimental data were compared to simulated tissue concentrations for which residual blood from the organ capillaries were taken into account. For both types of experiments, quantitative whole body autoradiography and wet-tissue dissection, a global fraction of residual blood was fitted to the experimental data assuming that the fraction is the same for every organ and each type of experiment. The fraction of residual blood is the ratio of blood volume in an organ contributing to the measured tissue concentrations to the total blood volume in the organ representing the in vivo blood contend. For tissue dissection studies the residual blood is the blood remaining in the harvested organ, for autoradiography studies it is the blood contribution which could not be excluded from image analysis. The assumption that the fraction of residual blood is the same for each organ was made to prevent parameter identification issues. Since the brain concentrations of 7E3 from [58] were corrected for residual blood, no residual blood was assumed for these data.

The following parameters were optimized globally, i.e., the same value was used in all simulations for all compounds and all species: *f*_*lymph*,*org*_ and *f*_*Jiso*,*org*_ for all 15 standard organs and the tumor, *k*_*up*_, *k*_*rec*_, the inter-species scaling exponent *γ*_*Jiso*_ and *k*_*ass*_ for FcRn binding. To improve identifiability *f*_*lymph*,*org*_ for liver and spleen and *f*_*Jiso*,*org*_ for small and large intestine were assumed to have the same value. The constant *k*_*ass*_ was assumed to be the same in acidic (endosomal space) and neutral environment (plasma/interstitial space). There were thus in total 30 organ specific parameters optimized and four further global parameters optimized across all species and compounds.

The concentration of free FcRn in the endosomal space was optimized species dependent, i.e., different values were allowed for mice, monkey and human. The following parameters were fitted specifically for individual compounds: the GFR fraction for the domain antibody and K_d_(FcRn) of BAY 79-4620 as well as the internalization rate constant of the BAY 79-4620–target complex. As mentioned above, two additional parameters, the fraction of residual tissue blood for autoradiography and for tissue dissection studies were fitted to the data.

The parameter estimation was performed using the Monte Carlo algorithm implemented in the Open Systems Pharmacology Suite. With that method, random permutations of the parameters are sequentially and randomly sampled. The root mean square error function was used.

### Data used for model evaluation

The following data sets were used to evaluate the model after parameter estimation.

#### Inulin in rats

The plasma and tissue concentrations after i.v. application of 20 and 200 mg/kg inulin in rats were taken from [61]. For the 200 mg/kg dose only plasma concentrations were reported.

#### Tefibazumab in humans

Tefibazumab is a humanized monoclonal antibody (IgG1). The target of the antibody is ClfA expressed by the bacterium *Staphylococcus aureus*. The plasma concentration–time profiles after single dose 15 min i.v. infusion of 2, 5, 10, or 20 mg/kg body weight in healthy adults were taken from [62].

### Software

PK-Sim version 6.3.2 [33–36] (www.systems-biology.com) was used to build the basic PBPK models. The model extensions for the protein model were implemented using MoBi version 6.3.2 [37]. Also the parameter estimation was performed in MoBi version 6.3.2. Plots were generated using MATLAB (version R2013b; The MathWorks, Inc., Natick, Massachusetts) by use of the MoBi Toolbox for MATLAB [37]. These software tools are available with version 7.0 under the name Open Systems Pharmacology Suite at www.open-systems-pharmacology.org. The present PBPK model is available in the Open Systems Pharmacology Suite from version 7.1 onwards.

## Results

The PBPK model for small molecules in PK-Sim was extended taking into account extravasation, transport of drug by lymph flow as well as endosomal clearance and recycling by FcRn as described above.

**Fig. 4:**
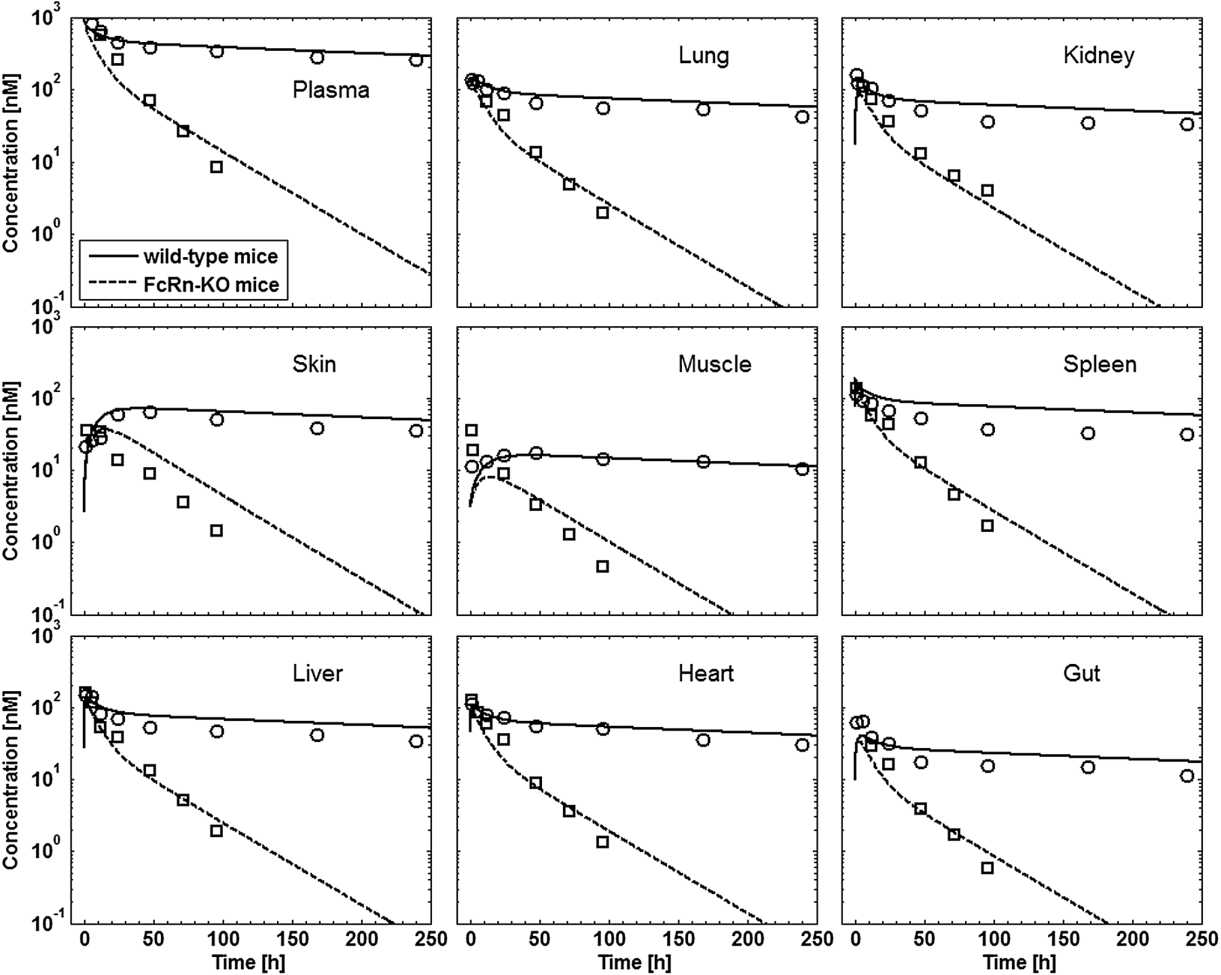
Comparison of simulated (lines) versus experimental (symbols) concentration–time profiles of the 7E3 antibody in wild-type (solid line, circles) and FcRn-knockout mice (dashed line, squares). Experimental data are taken from [27]

**Fig. 5:**
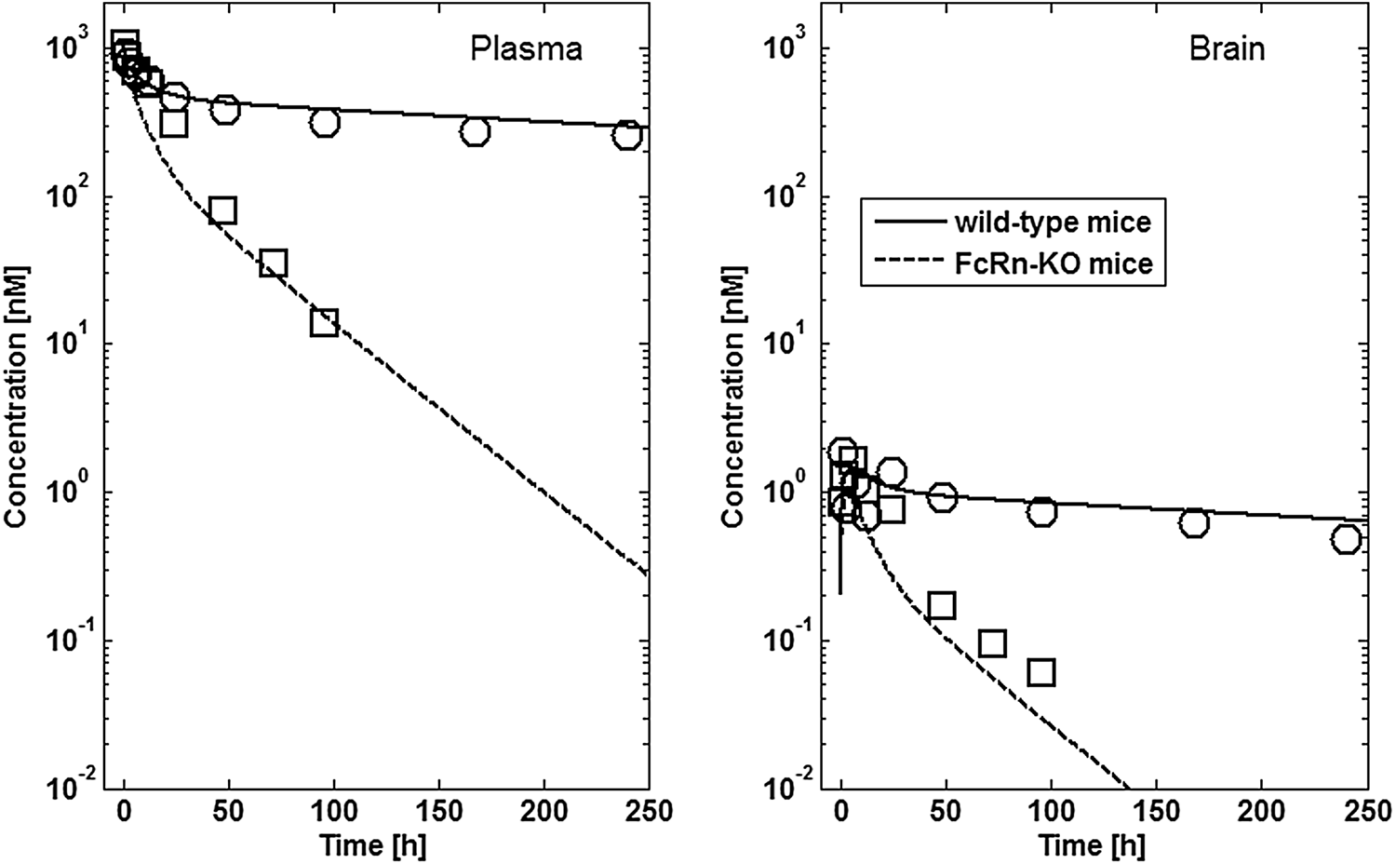
Comparison of simulated (lines) versus experimental (symbols) concentration–time profiles in plasma and brain tissue of the 7E3 antibody in wild-type (solid line, circles) and FcRn-knockout mice (dashed line, squares). Experimental data are taken from [58]

**Fig. 6:**
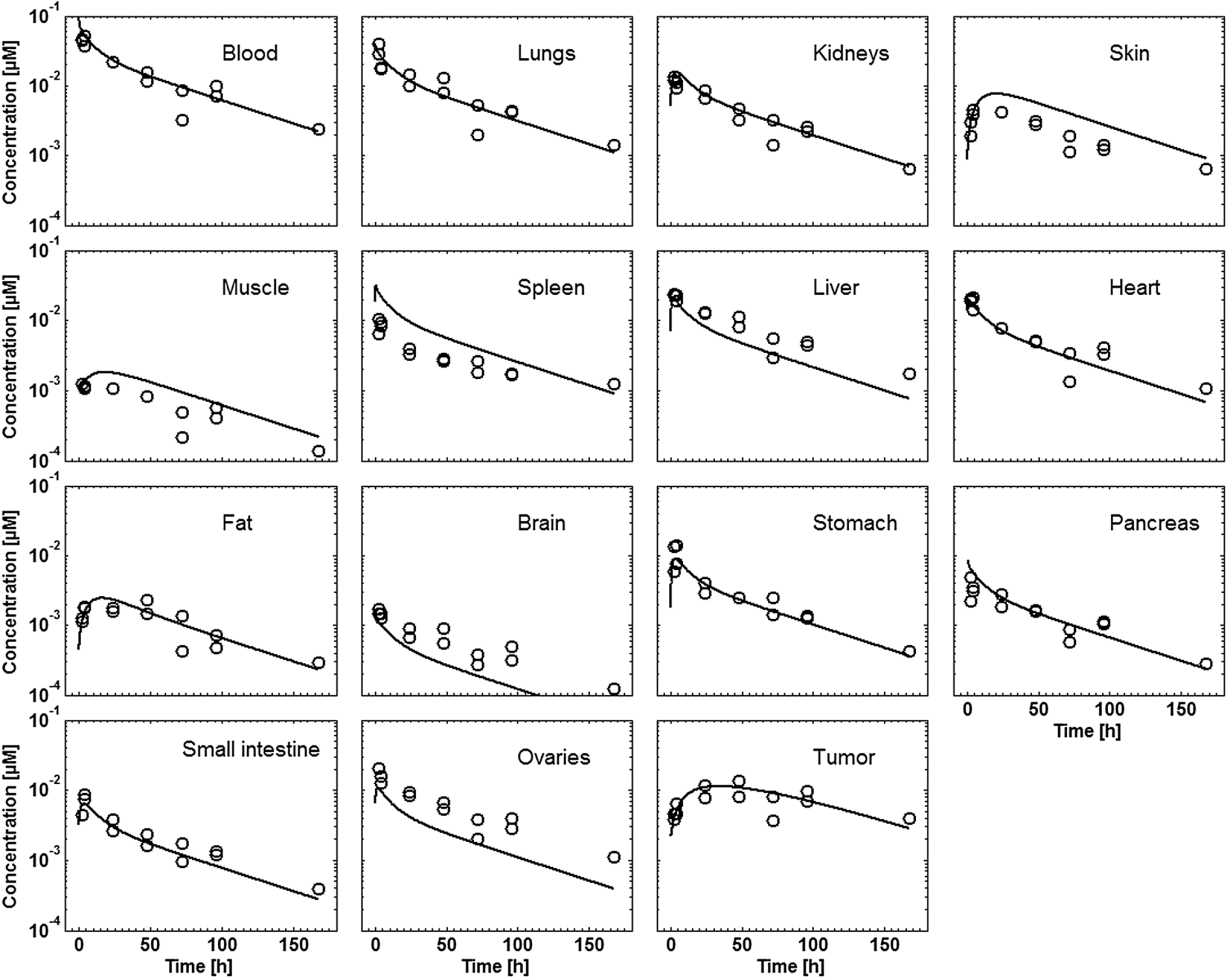
Comparison of simulated (lines) versus experimental (symbols) concentration–time profiles for BAY 79-4620 in mice. Experimental data from the autoradiography study, dose 1.25 mg/kg (in-house data)

**Fig. 7:**
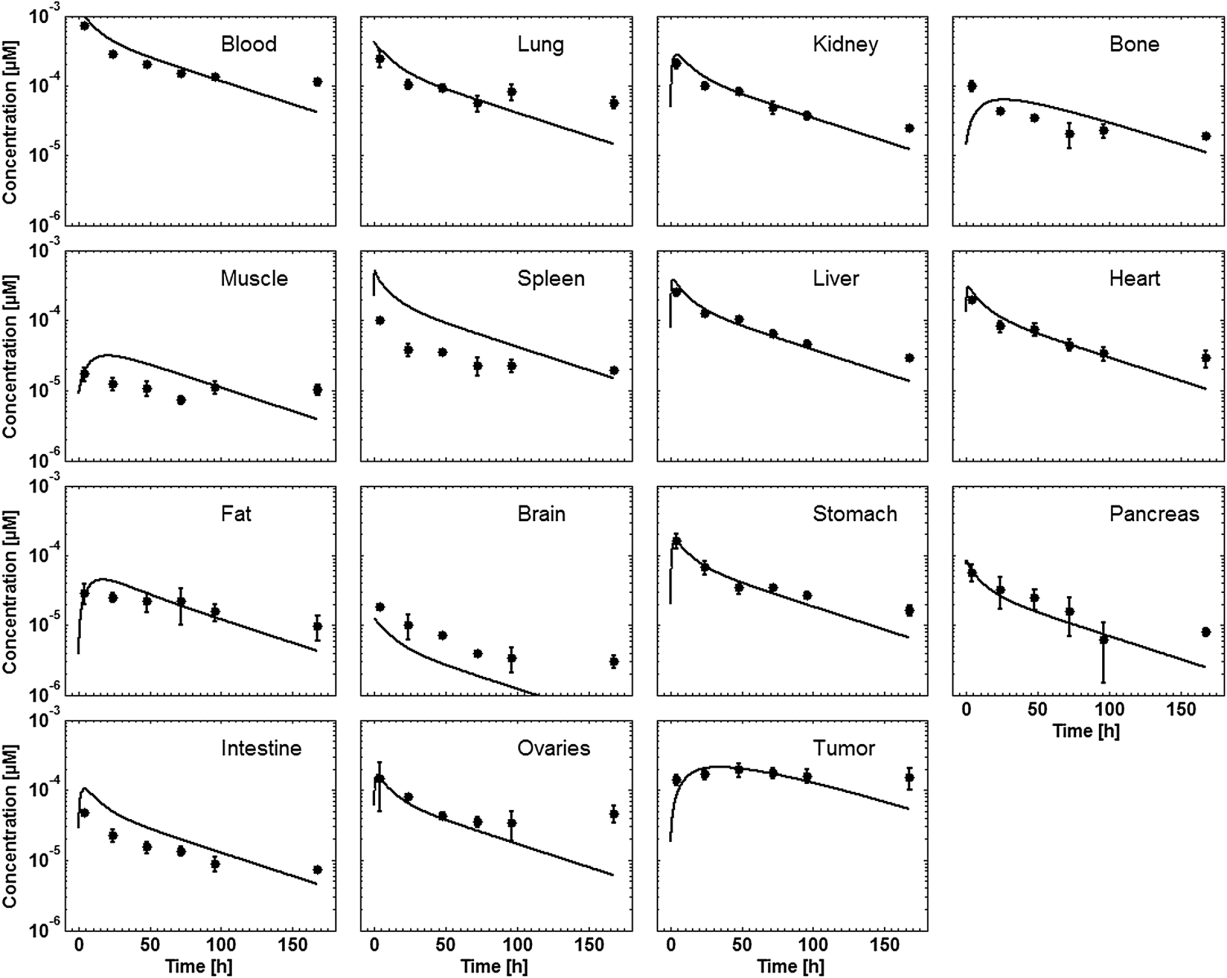
Comparison of simulated (lines) versus experimental (symbols) concentration–time profiles for BAY 79-4620 in mice. Experimental data from the tissue dissection study, dose 0.025 mg/kg (in-house data)

**Fig. 8:**
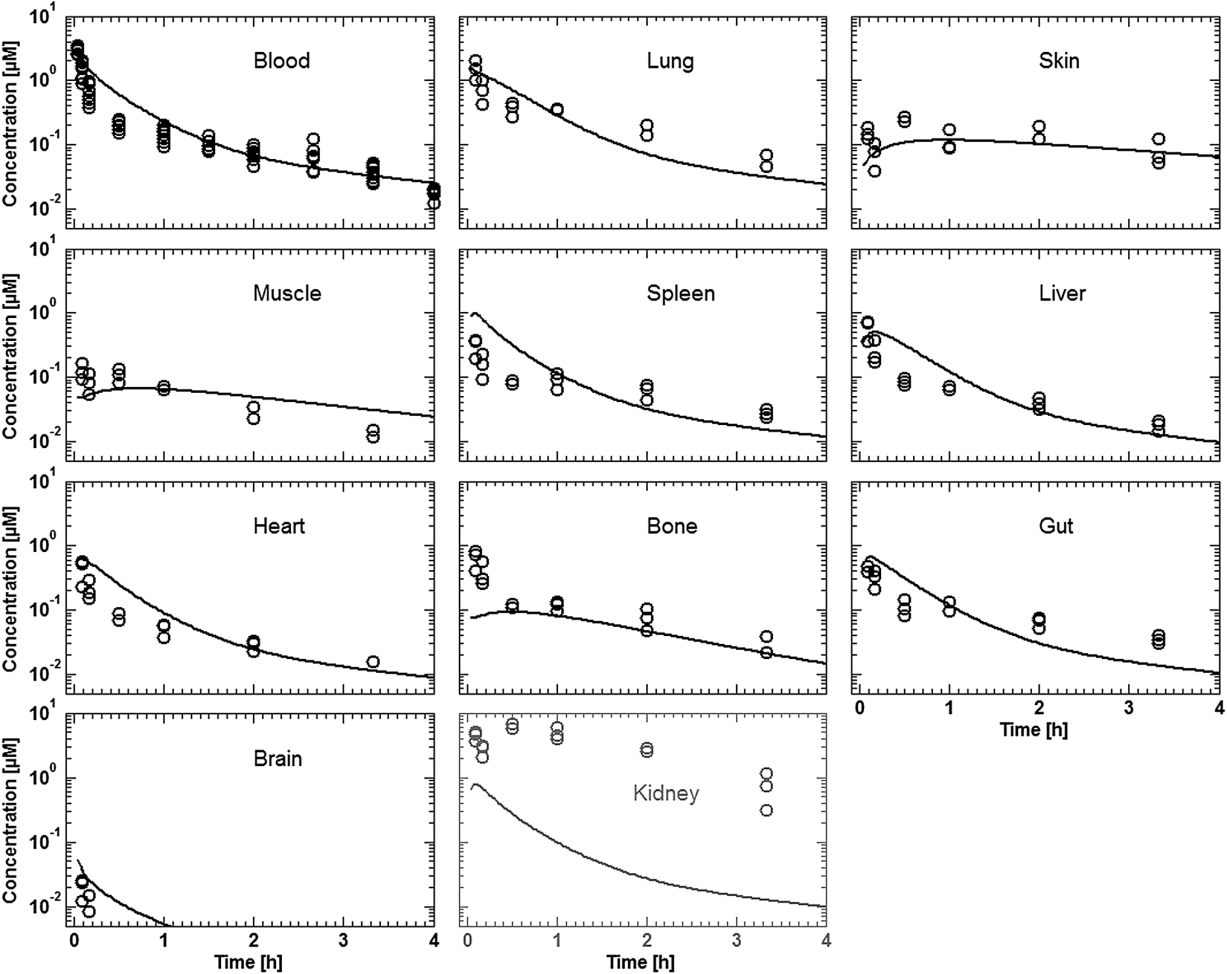
Comparison of simulated (lines) versus experimental (symbols) concentration–time profiles of the domain antibody dAb_2_ in mice. Experimental data are taken from [29]. Kidney data were not used during parameter estimation

**Fig. 9:**
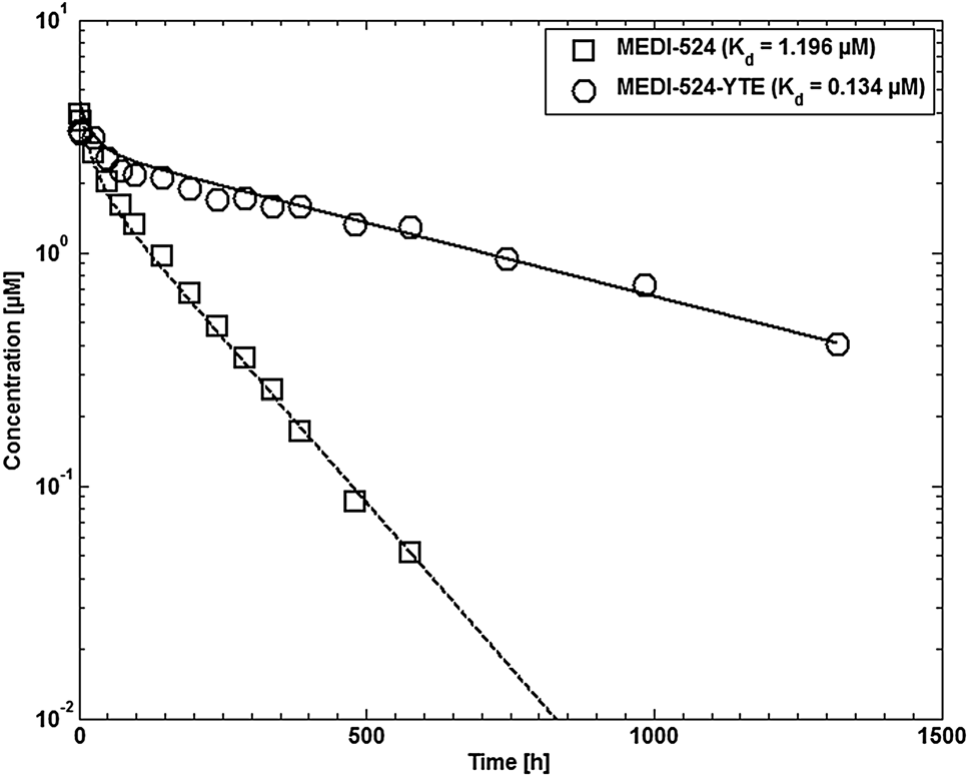
Comparison of experimental plasma concentration–time profiles for wild type MEDI-524 and the high affinity Fc variant MEDI-524-YTE in cynomolgus monkeys compared to simulation results. Experimental data are taken from [59]

**Fig. 10:**
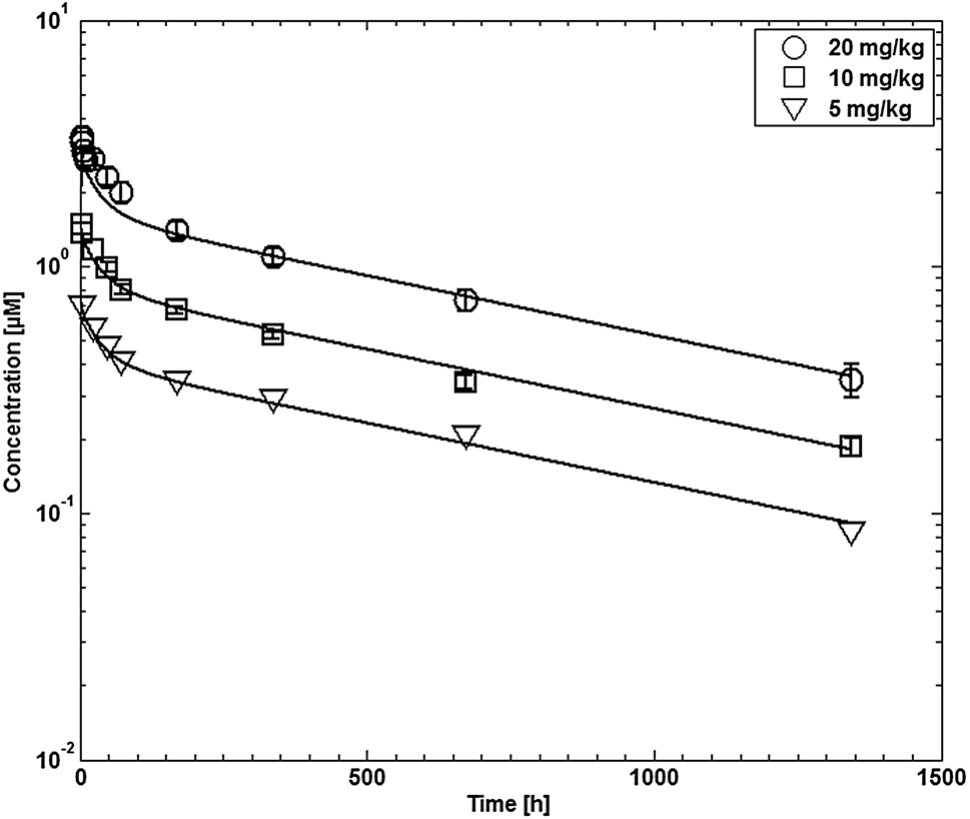
Comparison of experimental plasma concentration–time profiles for CDA1 in humans with simulation results. Experimental data are taken from [60]

**Fig. 11:**
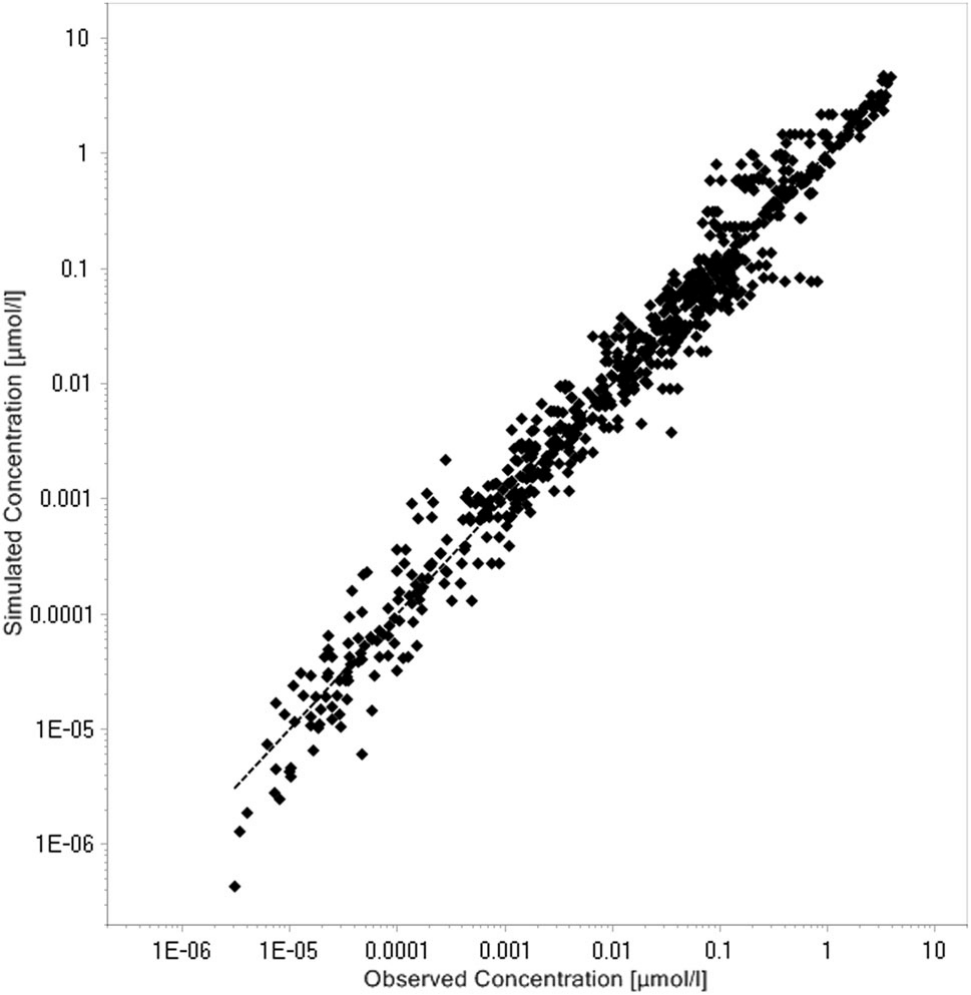
Simulated versus observed concentrations for all data used for parameter estimation

**Fig. 12:**
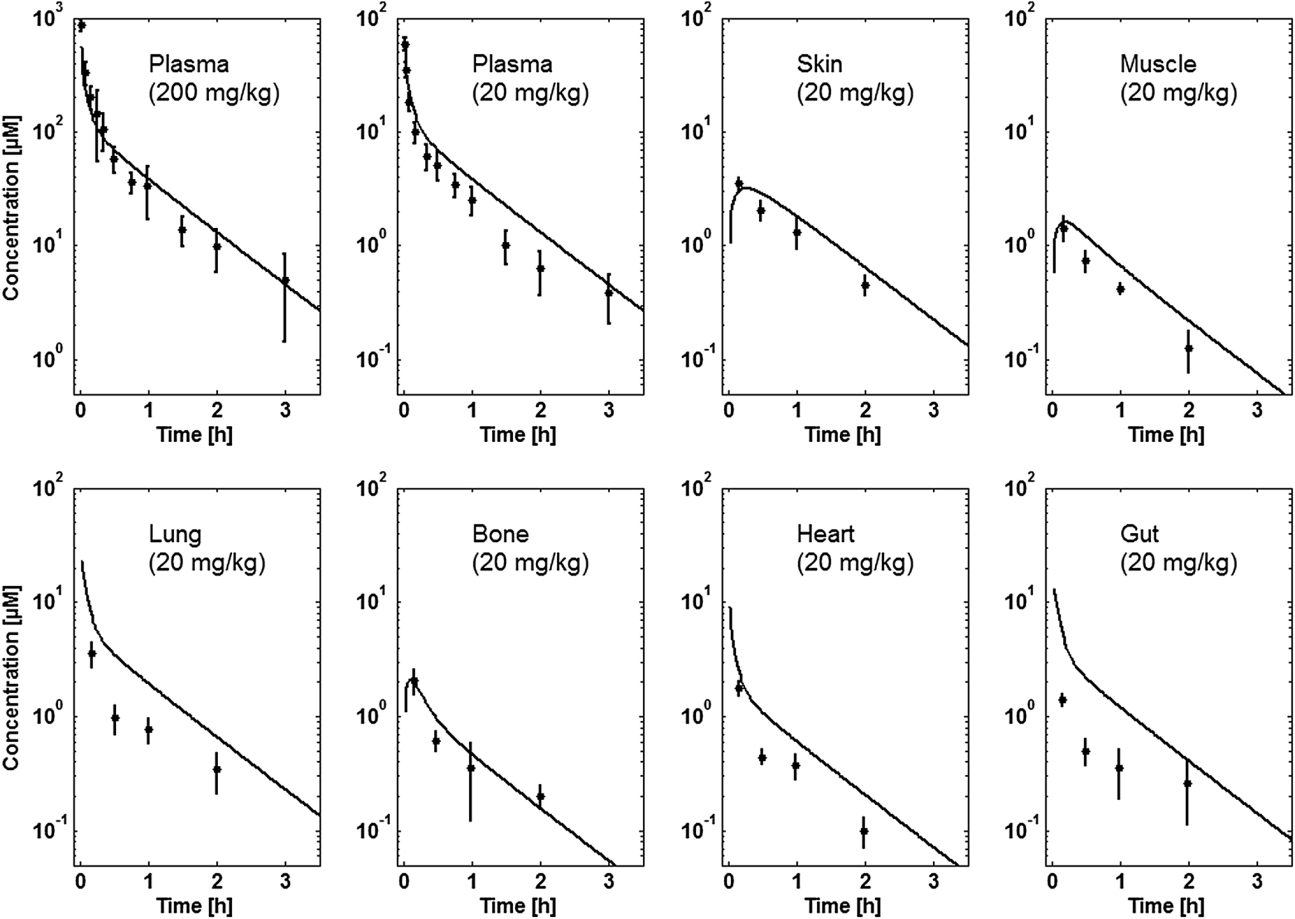
Comparison of experimental (symbols) and simulated (lines) plasma and tissue concentrations of inulin in rats for a dose of 20 and 200 mg/kg (plasma only). Experimental data are taken from [61]

**Fig. 13:**
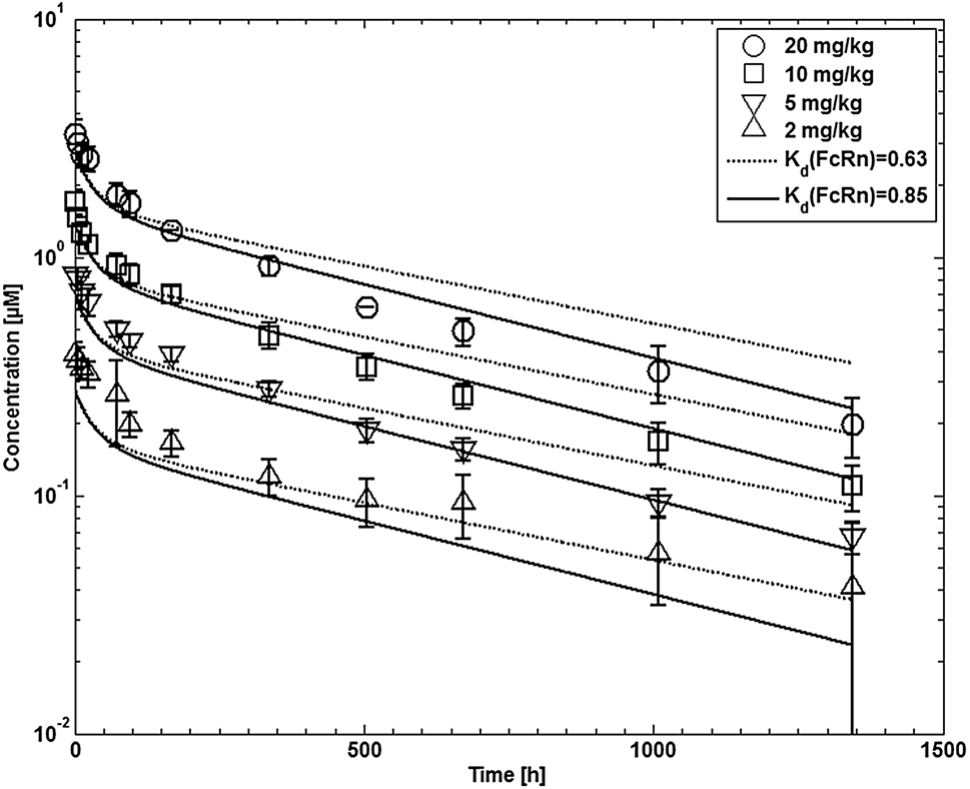
Comparison of experimental (symbols) and simulated (lines) plasma concentrations of tefibazumab in humans. Experimental data are taken from [62]. Dotted lines indicate predictions using the same affinity to FcRn as for CDA1 (0.63 µM). Solid lines indicate simulations using affinity to FcRn which was adapted to the experimental data (0.85 µM)

Tissue concentration–time profiles in mice for all represented tissues were used in order to identify lymph and recirculation flow rates (given as fraction of plasma and fluid flow via small pores, respectively). For this purpose, drugs with different solute radius were considered: the antibody 7E3 and the ADC BAY 79-4620 (solute radius 5.34 nm for both) and a domain antibody (solute radius 2.43 nm). Furthermore, plasma concentration–time curves for antibodies in monkeys (MEDI-524 and MEDI-524-YTE) and humans (CDA1) were considered to inform model parameters across different species. The only parameter informing species difference in drug distribution that was adjusted during parameter identification is the organ volume based allometric scaling exponent for the recirculation flow. Most relevant for the estimation of the parameters related to endosomal clearance and recycling by FcRn are the concentration–time profiles of 7E3 in wild type and FcRn knockout mice as well as MEDI-524 and its Fc variant MEDI-524-YTE (having an increased affinity to FcRn) in monkeys. The performance of the model for the different compounds and different species and the identified parameters are described in the following sections. The simulation results after parameter estimation are compared to the data used for fitting in Figs. 4, 5, 6, 7, 8, 9 and 10. All data are described reasonably well, the predicted versus observed concentrations for all data used for parameter estimation are shown in Fig. 11. The fractions of residual blood obtained from the parameter estimation were 42% for the autoradiography studies and 18% for the tissue dissection studies in line with data from literature [63]. The model was further evaluated by predicting the plasma PK of an additional antibody in humans (tefibazumab). Finally, the plasma and tissue concentration–time profiles for inulin were predicted and compared to experimental data in order to evaluate the model for a smaller macromolecule (solute radius 1.39 nm). These model evaluation results are given in Figs. 12 and 13.

### Fitting the PBPK model to mice data

Overall, the plasma and tissue data from the different mice studies are reasonably well described using lymph and recirculation flow rates which are consistent across the antibody and ADC as well as the smaller domain antibody (obtained from a global fit). The parameter values are given in Table 6. While the fluid recirculation flow fractions *f*_*Jiso*_ were estimated with very low coefficients of variation (CV < 1%), the CV are rather high for the lymph flow rate fractions *f*_*lymph*_ (CV between 17 and 50%, up to 91% for heart, cf. Tables S4, S5 in the supplemental material). From a sensitivity analysis (cf. Figs. S2, S3 of the supplemental material), it can be seen that the fluid recirculation flows are predominantly sensitive to AUC while the lymph flow rates are predominantly sensitive to the time of maximum concentration. A possible reason for the lower identifiability of the lymph flow rates is that the concentration profiles/time of maximum concentration is less well characterized by the experimental data than the AUC. The global parameters describing FcRn mediated clearance are given in Table 7. The CV for these parameters are low (CV < 1%, cf. Table S6 in the supplemental material). The value of the specific endosomal clearance rate constant calculated as difference *k*_*up*_ − *k*_*rec*_ is 0.205 min^−1^. This value is slightly smaller compared to values which had been previously obtained by fitting the endosomal clearance rate constant independently from uptake and recycling rate constants, 0.613 min^−1^ [25] and 0.715 min^−1^ [30].

**Table 6:** Lymph and recirculation flow factors obtained by parameter estimation

Organs	*f*_*lymph*_	*f*_*Jiso*_
Bone	6.62E−4	0.960
Brain	7.27E−5	0.404
Fat	7.54E−3	0.357
Gonads	1.11E−2	0.960
Heart	1.47E−3	0.960
Kidney	7.09E−4	0.761
Large intestine	1.44E−2	0.179
Liver	1.99E−2	0.960
Lung	3.56E−5	0.010
Muscle	2.01E−3	0.292
Pancreas	3.03E−2	0.010
Skin	3.52E−3	0.617
Small intestine	1.95E−3	0.179
Spleen	1.99E−2	0.010
Stomach	2.04E−3	0.960
Tumor	3.65E−3	0.281

**Table 7:** Endosomal clearance/FcRn related parameters obtained by parameter estimation

Free endosomal FcRn concentration in mice (µmol/l)	38.7
Free endosomal FcRn concentration in monkeys (µmol/l)	21.0
Free endosomal FcRn concentration in humans (µmol/l)	80.8
Rate constant for endosomal uptake, k_up_ (min^−1^)	0.294
Rate constant for endosomal recycling, k_rec_ (min^−1^)	0.0888
Association rate constant for FcRn binding, k_ass_ (l/µmol/min)	0.87

The difference in PK of the antibody 7E3 for wild type mice compared to FcRn knockout mice is well represented by the model (cf. Figs. 4, 5). Also the relative tissue concentrations (considerably lower brain concentrations, cf. Fig. 5, and slightly lower muscle concentrations, cf. Fig. 4, as compared to other tissues) are described well by the model. Tissue concentrations tend to be overestimated by the model especially for the skin concentrations in the FcRn KO mice and the spleen concentrations in the control mice. The initial concentrations in muscle, especially for the FcRn KO mice, and in gut are underpredicted.

The PK of BAY 79-4620 is also well described by the model (cf. Fig. 6 for the autoradiography study and Fig. 7 for tissue dissection study). The terminal half-life of BAY 79-4620 (~ 48 h) is considerably shorter than that of other human or humanized antibodies in mice [64]. Since BAY 79-4620 shows a high clearance, the affinity to FcRn was fitted to the experimental data. A value of 12.7 µM was obtained after parameter estimation. This value might reflect an altered affinity to FcRn due to the conjugation of the toxophore. Alternatively, the low affinity could be a surrogate for a clearance process not represented in the model. For the internalization rate constant of the BAY 79-4620–target complex, a value of 0.027 min^−1^ was obtained. The tissue concentrations from the low dose tissue dissection study (dose approximately 0.025 mg/kg) are similarly well described as the tissue concentrations from the autoradiography study (dose 1.25 mg/kg), with the exception of the late concentrations at 168 h after administration from the tissue dissection study which are underestimated by the model.

The simulated concentration–time profiles of the domain antibody in mice are compared to the experimental data in Fig. 8. Overall, the experimental data are reasonably well described. Only the kidney concentrations are significantly underestimated by the simulation. However, this was expected as the kidney model structure does not represent tubular fluid und contributions from tubular fluid and re-absorption to tubular walls after renal clearance are not taken into account. Consequently, the kidney data were not used during parameter estimation. The decrease of blood concentration in the initial distribution phase is slightly underestimated by the model. The slow tissue uptake in skin and muscle is represented well by the model, while the initial spleen and bone concentrations are overestimated.

For the glomerular filtration coefficient a value of 0.24 was obtained which is slightly smaller than the value of 0.34 estimated from the relationship with molecular size given in [65].

### Fitting the PBPK model to data from monkeys and humans

Following the description of the PBPK simulations in mice, the results for the protein PK in monkeys and humans are considered in the following section. The best value for the inter-species scaling exponent of the fluid recirculation flow, *γ*_*Jiso*_ was 2/3. This value was chosen in the final model since it was slightly superior regarding the distribution behavior in humans compared to an exponent 3/4 which is commonly used, e.g., for cardiac output [66].

The simulated antibody plasma concentrations in monkeys and humans are compared to experimental data in Fig. 9 (monkeys) and Fig. 10 (humans), respectively. The differences in clearance for MEDI-524 and the Fc variant MEDI-524-YTE is well represented by the simulations using the different experimental affinities to FcRn. The fitted parameters relevant for endosomal clearance are given in Table 7.

The simulated plasma concentrations for CDA1 in humans are compared to experimental data used for fitting in Fig. 10. The initial plasma concentrations are slightly underestimated especially for higher doses; however the overall agreement with the experimental data is good.

### PBPK model evaluation

The final protein PBPK model was further evaluated with inulin, which has a considerably smaller solute radius than the proteins considered before. Thus, the extravasation is considerably faster and extravasation is almost exclusively determined by diffusion and not convection for most organs (cf. supplemental material, Table S9). The simulations results are compared to experimental data in Fig. 12. Overall, the experimental and tissue concentrations of inulin are predicted well. The gut, lung and heart concentrations are overestimated by the model. A possible reason might be a slight underestimation of the plasma–interstitial exchange rate.

For humans an additional dataset of plasma concentrations of an antibody with no endogenous target, tefibazumab, was used for model evaluation. The simulation results using the same model parameters as obtained by the parameter estimation are compared to experimental data in Fig. 13. The distribution behavior of tefibazumab is similar to that of CDA1 and is correspondingly similarly well described by the model. However, the clearance of tefibazumab seems to be slightly higher which might be due to a slightly different affinity to FcRn. Thus, with the standard affinity used for human antibodies (K_d_ = 0.63 µM) the clearance is slightly underestimated. After manually adapting the affinity to FcRn (K_d_ = 0.85 µM) the simulation results are in good agreement with the experimental data, except for the lowest dose.

## Discussion

A PBPK model for therapeutic proteins was developed taking into account the general processes of extravasation, transport of drug by lymph flow as well as endosomal clearance and recycling by FcRn. The physiological parameters used to describe extravasation are the properties of the vascular walls as well as lymph and the fluid recirculation flow rates. While the properties of the vascular walls (cf. Table 1) were taken from the literature assuming two types of capillaries, the lymph and the fluid recirculation flow rates were estimated using plasma and tissue concentration–time profiles from compounds with different solute radius (5.34 and 2.43 nm). Model predictions employing this parameterization were evaluated using also inulin having a smaller radius of 1.39 nm. It was thus shown, that the model is able to describe the passive distribution behavior, which is determined by the interplay of extravasation and the transport from interstitial space back to the circulation by lymph flow, for macromolecules with a wide range of molecular size. For the estimation of the physiological parameters related to the second additional mechanism considered in the model, endosomal clearance and recycling by FcRn (cf. Table 7) the concentration–time profiles of the antibody 7E3 in wild type versus FcRn knockout mice as well as concentration–time profiles of an antibody and its Fc variant having an increased affinity to FcRn in monkeys (MEDI-524 and MEDI-524-YTE) were most relevant. The generic model in PK-Sim is thus able to describe the generally relevant processes of passive distribution and clearance of therapeutic antibodies. Further processes which are more drug specific, e.g., target binding and target mediated clearance, can be added by the user as needed for a given therapeutic protein.

Regarding the description of extravasation, different variants were used in previously published PBPK models, considering single or two pore types, including recirculation flow or not, considering both, convection and diffusion or convection only, cf. [7] for a review.

In the current model, the two-pore formalism as described by Rippe and Haraldsson [45, 46] was used to represent extravasation (Eq. 2). Molecules can pass through the pores by convection as well as diffusion. Convection is predominant for large proteins like antibodies and diffusion for small fragments or small peptides (cf. supplemental material, Tables S7, S8, S9). The lymph flow rates for the different organs, given as fraction of plasma flow, were fitted to tissue concentration–time profiles of antibodies and an antibody fragment (domain antibody). The resulting lymph flows range from 0.066 to 3% of plasma flow for most organs aside from brain (0.0073%) and from lung (0.0036%), for which the fraction refers to the total cardiac output plasma flow. These values are similar to values used in previous PBPK models reported by Sepp et al. [29] (0.002–1.2%), Shah and Betts [30] (0.2% for all organs), and Garg and Balthasar [27] (2–4%). The total lymph flow (sum over all organs) in the current model is 0.4% of the total plasma flow in good agreement with the 0.2–0.3% estimated for the total afferent lymph low in human [67].

In contrast to the PBPK model of Sepp et al. [29], the permeability–surface area products are not set proportional to the lymph flow but are calculated from vascular properties and the solute radius of the drug. The organs in the present model were classified into two different types reflecting the properties of the vascular endothelium (pore radii and fraction of flow via large pores). In one class, the endothelial properties correspond to continuous (non-fenestrated and fenestrated) endothelium, in the other class they correspond to discontinuous endothelium [49–51]. In the present model, liver and spleen were assigned to have discontinuous capillaries while all other organs were assigned to have continuous capillaries [51]. Physiologically, in bone both capillary types are present, continuous endothelium in cortical bone and discontinuous in bone marrow [51]. This suggests an explicit, separated representation of cortical/trabecular bone and bone marrow in a future extension of the model. In the present model the bone was treated as one organ having continuous endothelium.

Several specific mechanisms have previously been discussed to explain the low brain/plasma concentration ratios observed after antibody application, including restricted paracellular transport across brain capillaries, convective flow of central nervous system fluids, and receptor-mediated efflux across brain capillaries [58, 68, 69]. In the present model, brain was treated as a normal organ which was fitted to brain tissue data [58] corrected for residual blood contribution, which is important due to the low antibody concentrations in brain. The low brain concentrations in the present model arise from a slow brain uptake due to a low lymph flow and a low hydraulic conductivity.

In the present PBPK model the kidney has the same organ model structure as other organs. Thus small proteins are considered to be cleared after glomerular filtration in the kidney and drug within the tubular fluid does not account to total kidney concentrations as it was considered in the PBPK model by Sepp et al. [29]. Also, reabsorption by the tubular wall and catabolism in tubular cells [70] was not taken into account.

As described above, the distribution behavior of the drug in the present PBPK model is represented by the interplay of extravasation and transport of drug from interstitial space back to the circulation in absence of further mechanisms like target mediated deposition. The only drug specific parameter relevant for drug distribution considering the described generic processes is the solute radius. In principle, charge does also influence extravasation and distribution but its effect is not consistently described for the different organs [45, 46, 71, 72]. Charge effects are thus not explicitly taken into account in the present model.

The sub-model to describe the endosomal clearance and FcRn mediated recycling used in the present study is similar to that reported by Garg and Balthasar [27] with the main difference that the drug–FcRn binding reaction is explicitly represented in a simplified sub-model. This allows specifying different FcRn binding affinities for the drug and the endogenous IgG. A further difference is that the binding is represented in the acidic endosomal space as well as in the neutral environment. In the present study, the K_d_(FcRn) value in the neutral environment was set to a high value representing virtually no binding which is usually reasonable for wild type antibodies [54]. However, for engineered antibodies, increased binding at neutral pH seems to be able to counteract the half-life extending effect of high affinity binding at acidic pH [73, 74]. Recently, a mechanism-based based model focusing on the effect of FcRn binding on antibody pharmacokinetics was published Ng et al. [32]. Taking into account the return of the drug–FcRn complex into the endosomal space, this model was able to describe the effect from different FcRn affinities in endosomal and neutral environment on the PK of antibodies.

The model developed in the present study was able to describe the different clearance in wild type and FcRn knockout mice and the different clearance of MEDI-524 and its high Fc affinity variant MEDI-YTE very well.

The value of the association rate constant for FcRn binding obtained by parameter estimation was 0.87 l/µmol/min which is lower than typical measured in vitro values (~ 7–40 l/µmol/min) [75]. This could reflect that processes like the return of the drug–FcRn complex into the endosomal space [32] or endosomal trafficking [20] are missing in the present model.

For the model development, the K_d_(FcRn) values of the antibodies were taken from different sources but all values originate consistently from assays using immobilized antibody and 1:1 stoichiometry for the data analysis. Experimental K_d_(FcRn) can vary considerably for different assays [76, 77]. The fitted endosomal FcRn concentrations depend on the K_d_(FcRn) values used as input parameters. Thus, it should be noted that, when simulating a new drug, the K_d_(FcRn) values used should be consistent with those used to estimate the endogenous FcRn concentrations. Establishing an in vitro to in vivo correlation for a certain assay as it was done by Ng et al. [32] is a possible solution to this challenge. Note, that the in vitro to in vivo correlation used by Ng et al. [32] is linear for the affinity in the acidic endosomal space, while it is nonlinear for the affinity at physiological pH in order to explain the PK of several Fc variants of an antibody.

The FcRn concentration is assumed to be the same in each organ and the endosomal uptake is proportional to the endosomal volume which in turn is proportional to the vascular volume in each organ. In the current model muscle (large organ) and liver (relatively large organ with relatively large vascularization) contribute most to total antibody clearance. Both organs are known to be major sites of antibody catabolism [78, 79]. For a refinement of the quantitative organ contribution to antibody clearance, the bio-distribution data of ^111^In-labeled antibodies, indicating cumulative tissue uptake of antibodies and metabolites, could be used [24, 80]. A PBPK model taking into account tissue specific FcRn expression can be found in [20].

While the model structure allows a drug to enter the endosomal space from plasma as well as from interstitial space, the parameterization in the present model was chosen such that drug enters the endosomal space exclusively from plasma and also that the drug–FcRn complex recycles exclusively to plasma ($f_{vas}^{up}$ and $f_{vas}^{rec} = 1$). With this parameterization no net extravasation via the endosome, i.e., no transcytosis across the capillary walls is taken into account in the model. The relative contribution of convection via large pores and transcytosis is controversially discussed [81–83]. While there is evidence for transcytosis, the fractions of endosomal uptake and recycling from and to plasma and interstitial space do not agree across published PBPK models. Garg and Balthasar [27] assume an equal rate constant for endosomal uptake and fitted a fraction of 0.715 for recycling to plasma, a value which was also used by Shah and Betts [30]. Chabot et al. [19] fitted an almost exclusive uptake from plasma (fraction 0.971) and recycling predominantly to interstitial space (fraction 0.364 for recycling to plasma). Ferl et al. [25] and Davda et al. [23] assume uptake and recycling solely from and to plasma. By choosing $f_{vas}^{up}$ and $f_{vas}^{rec} = 1,$ the same assumption is made in the present model leading to a clear separation of the mechanism for extravasation described by the two-pore equation (2) and endosomal clearance/FcRn mediated recycling. To allow future evaluation with additional data using a different parameterization, the extended structure was chosen.

The model structure used for the endosomal clearance and FcRn mediated recycling is not specific for endogenous IgG. Since albumin is binding independently from endogenous IgG [84], the model can be recalibrated using endogenous albumin instead of endogenous IgG in order to describe the half-life extension of albumin fusion proteins [85].

Only the FcRn binding model involves parameters which are explicitly species dependent. These parameters were fitted to PK data for mice, monkey and human in the current model. The parameters describing extravasation and lymph flow are either assumed to be species independent or scale with known physiology. They can thus be used for all animal species and were evaluated in the current study for mice, rats, monkeys and humans comprising a large body size range.

Besides i.v. dosing, subcutaneous dosing is a common application route for therapeutic proteins. PBPK models including a subcutaneous dosing site have been recently published [28, 39, 86]. These or similar extensions can also be made for the present PBPK model in order to describe the PK after subcutaneous application. An application compartment can be added using the software MoBi (http://open-systems-pharmacology.org).

## Conclusions

A PBPK model for protein therapeutics representing the general mechanisms driving the distribution and clearance of large molecules was developed. For model development and evaluation, compounds with a wide range of solute radius (1.39–5.34 nm) were used. It was possible to describe passive antibody distribution by extravasation and lymph flow for small to large species (mouse, monkey and human) assuming the lymph flow to be proportional to the plasma flow and assuming an organ volume specific allometric scaling for the recirculation flow being proportional to lymph flow. Also, endosomal clearance and recycling by FcRn are represented by the model and were parameterized for mouse, monkey and human. The implemented model is available in the Open Systems Pharmacology Software Suite (www.open-systems-pharmacology.org) [37]. The functionality of the software platform allows custom-made extensions of the model to reflect missing mechanisms relevant to describe the PK of a given therapeutic protein, e.g., target binding and target mediated clearance. Furthermore, the expression database allows the analysis of relative on-target (e.g., tumor) PK/PD effects versus off-target toxicity. The model is an extension of the small molecule model in PK-Sim, keeping the same model structure and organ representation. It is thus especially well-suited to simulate small and large molecules in a single PBPK framework which is, for example, important for the simulation of ADCs with an explicit representation of the ADC and toxophore [87] or to simulate PK/PD effects of combination therapies including small and large molecules.

## Electronic supplementary material

Below is the link to the electronic supplementary material.

Supplementary material 1 (TXT 2013 kb)

Supplementary material 2 (PDF 183 kb)
