# Supplementary material for: A generic whole body physiologically based pharmacokinetic model for therapeutic proteins in PK-Sim
Source: J Pharmacokinet Pharmacodyn. 2017 Dec 12;45(2):235–57. doi: 10.1007/s10928-017-9559-4 (PMC5845054; doi:10.1007/s10928-017-9559-4)
Supplement: Supplementary file 2 — Supplementary material 2 (PDF 183 kb) [file 10928_2017_9559_MOESM2_ESM.pdf]

A generic whole body physiologically based pharmacokinetic model for therapeutic proteins in PK-Sim

Supplementary Information

Christoph Niederalt, Lars Kuepfer, Juri Solodenko, Thomas Eissing, Hans-Ulrich Siegmund, Michael Block, Stefan Willmann, Jörg Lippert

Contents

1. Hydrodynamic radius vs. molecular weight 1
2. Experimental vs. model values for permeability-surface area products and surface areas for the exchange across capillaries 3
3. Sensitivity and confidence intervals of PBPK model parameters obtained by parameter estimation 5
4. Contribution of convection and diffusion to extravasation 10
5. Equations for steady state concentrations for FcRn and endogenous IgG 12
References 15

1. Hydrodynamic radius vs. molecular weight

An empirical relationship for the dependency of the hydrodynamic radius a_e of proteins on the molecular weight was established by a least squares fit of a and b in the linear equation

$$\log 10(a_e) = a \cdot \log 10(molecular\ weight) + b \qquad (1)$$

to the data collection given in Table S1.

The fitting (cf. Figure S1) resulted in Equation (2)

$$a_e = 0.0333 \cdot molecular\ weight^{0.4226} \qquad (2)$$

with a_e given in [nm] and molecular weight given in [g/mol].

For comparison of this equation to similar equations see Ref. [1]. Similar equations can be found also in Refs. [2,3]. The hydrodynamic radius for the domain antibody dAb_2 used by Sepp et al. [2] and obtained with their equation was 2.49 nm. This value is very similar to the value used in the present study (2.43 nm) obtained by Equation (2).

Table S1: Data used to fit the equation describing the relationship of hydrodynamic radius to molecular weight. The hydrodynamic radius calculated

Name	MW [g/mol]	radius [nm]	Data source
Prealbumin	55000	3.25	Reference a)
Antichymotrypsin	68000	3.42	Reference a)
Hemopexin	57000	3.5	Reference a)
Albumin	66300	3.58	Reference a)
Transferrin	76500	3.67	Reference a)
IgG	150000	5.34	Reference a)
IgA	160000	5.68	Reference a)
Macroglobulin	725000	9.35	Reference a)
IgM	950000	12.1	Reference a)
Inulin	5500	1.5	Reference b)
Myoglobin	17000	1.9	Reference b)
Albumin	69000	3.55	Reference b)
Fab	48000	3	Reference c)
Lysozyme	14700	1.9	Reference d)
Chymotrypsinogen	25000	2.4	Reference d)
Insulin [e)]	34200	2.7	Reference d)
Ovalbumin	43000	3	Reference d)
Hexokinase	102000	4.3	Reference d)
Apoferritin	443000	8.2	Reference d)
Thyroglobulin	669000	10.1	Reference d)

a) Ref. [4], Table 4; b) Ref. [5]; c) Ref. [6]; d) [7] e) The compound reported as insulin is probably insulin hexamer according to the molecular weight.

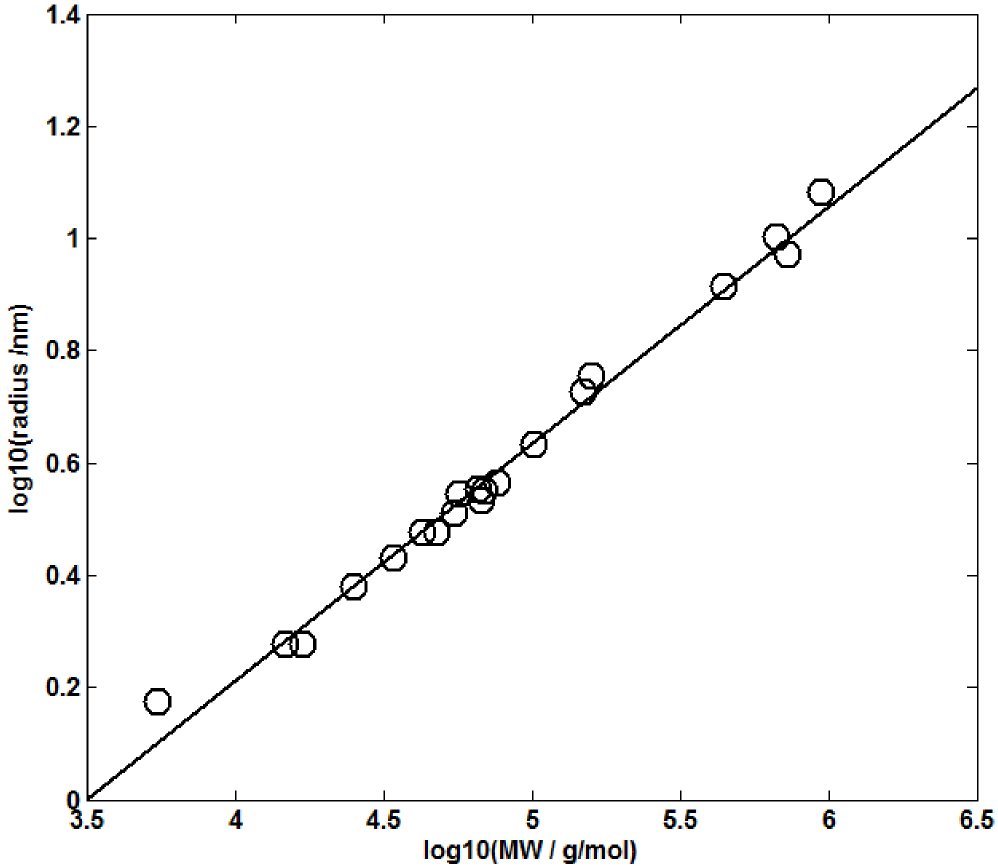

Figure S1: Relationship between molecular size and molecular weight for data in Table S1 (symbols) and Equation (1) with a= 0.4226 and b= -1.4775 (line).

2. Experimental vs. model values for permeability-surface area products and surface areas for the exchange across capillaries

Permeability-surface area products (PS) for the exchange across capillaries calculated using the equations

$$S_{org} = k \cdot f_{vas,org} \cdot V_{org} \text{ and}$$

$$P_{S,org} = \xi_{S,org} \cdot \frac{D}{L} \frac{A_{S,org}}{S_{org}}, \; P_{L,org} = \xi_{L,org} \cdot \frac{D}{L} \frac{A_{L,org}}{S_{org}}$$

used in the PBPK model are compared to experimental data in Table S2 for inulin. Calculated capillary surface areas are compared to experimental data in Table S3.

Table S2: Comparison of experimental permeability-surface area products (PS) for trans-capillary exchange of inulin to values calculated by the equations implemented in the PBPK model.

	Experimental PS for inulin[a)] [ml/min/100 g]	Calculated PS for inulin[b)] [ml/min/100 g]
Muscle (skeletal)	0.59 – 1.62	0.96
Heart	7.8 – 19.2	15.4
Intestine	23	9.8 (small intestine);<br>7.5 (large intestine)
Lung	22.8 - 90	14.5
Kidney	1200 (peritubular capillaries)	53.7

a) Experimental values from different studies reviewed in Ref. [8].
b) Sum of small and large pore contributions ($P_LS + P_SS$). The small pore contribution is larger than 99%.

Table S3: Comparison of experimentally estimated capillary surface areas to values calculated by the equation implemented in the PBPK model.

	Experimental capillary surface area [a)] [cm^2 / g]	Calculated capillary surface area [cm^2 / g]
Muscle (skeletal)	62 - 240	24.7
Brain (cortex)	130 - 150	35.1
Heart	500 - 575	249
Lung	3000 - 3500	595
Kidney	350 - 1200 (peritubular capillaries)	100

a) Experimental values from different studies reviewed in Ref. [8].

3. Sensitivity and confidence intervals of PBPK model parameters obtained by parameter estimation

For the parameters of the generic PBPK model obtained by parameter identification a sensitivity analysis was performed by calculating the sensitivity coefficients $S_{i,j}$ of PK parameters PK_j to the PBPK model parameters p_i using the equation

$$S_{i,j} = \frac{\Delta PK_j}{\Delta p_i} \cdot \frac{p_i}{PK_j}.$$

The sensitivities coefficients are shown in Figure S2 (AUC) and Figure S3 (t_{max}) for the lymph flow (f_{Lymph}) and fluid recirculation flow (f_{Jiso}) proportionality factors for each organ and in Figure S4 for endosomal clearance / FcRn related parameters.

The 95% confidence intervals (95%CI) and coefficients of variation (CV) calculated from the confidence intervals by CV (%) = 95%CI/1.96/parameter value*100 of the optimized physiological parameters are given in Table S4 to Table S6.

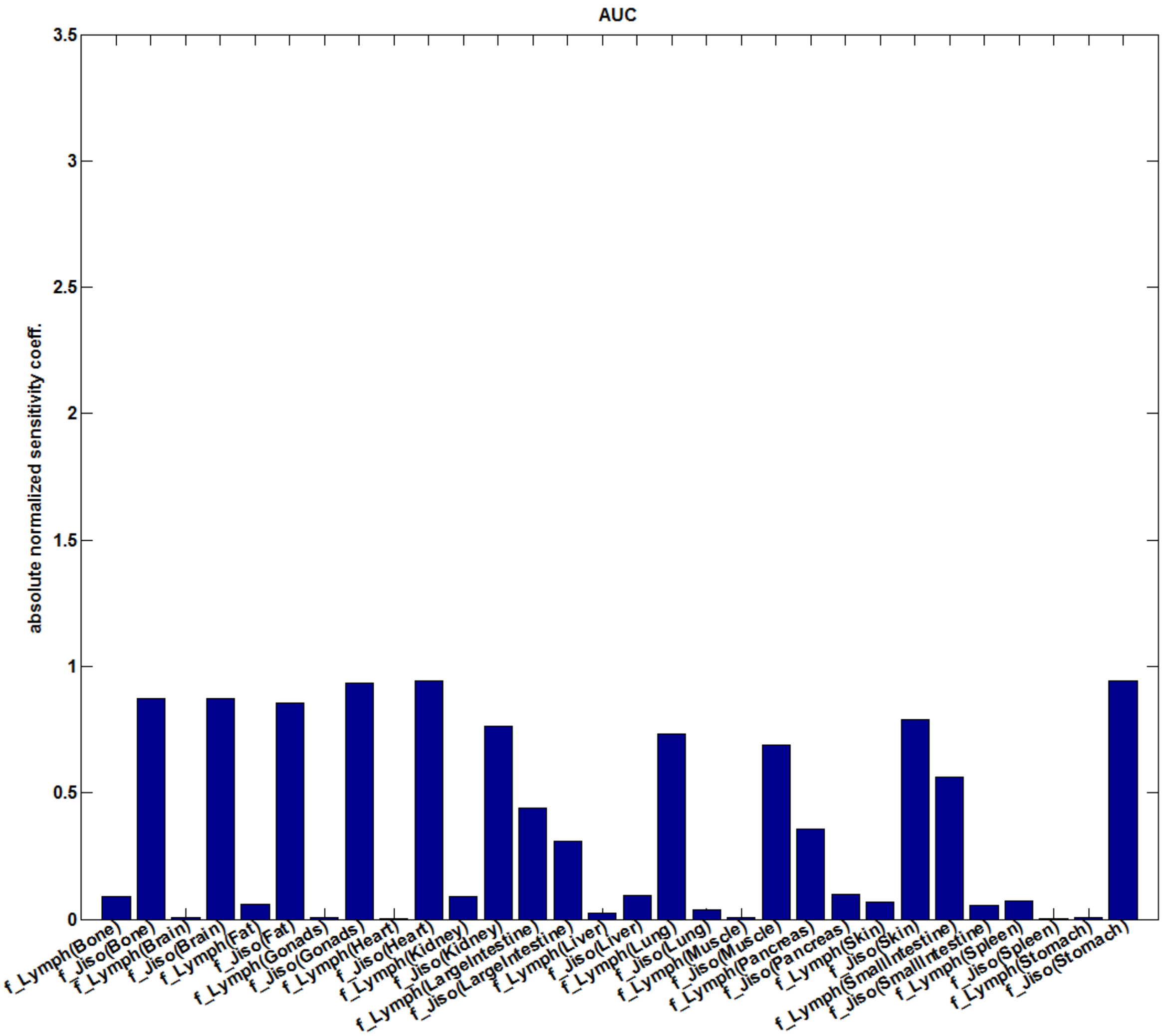

Figure S2: Sensitivity coefficients for the AUC of the tissue concentrations of the respective organs for an antibody in mice (7E3 in wild type mice) for lymph (f_Lymph) and fluid recirculation flow (f_Jiso) proportionality factors of the different organs.

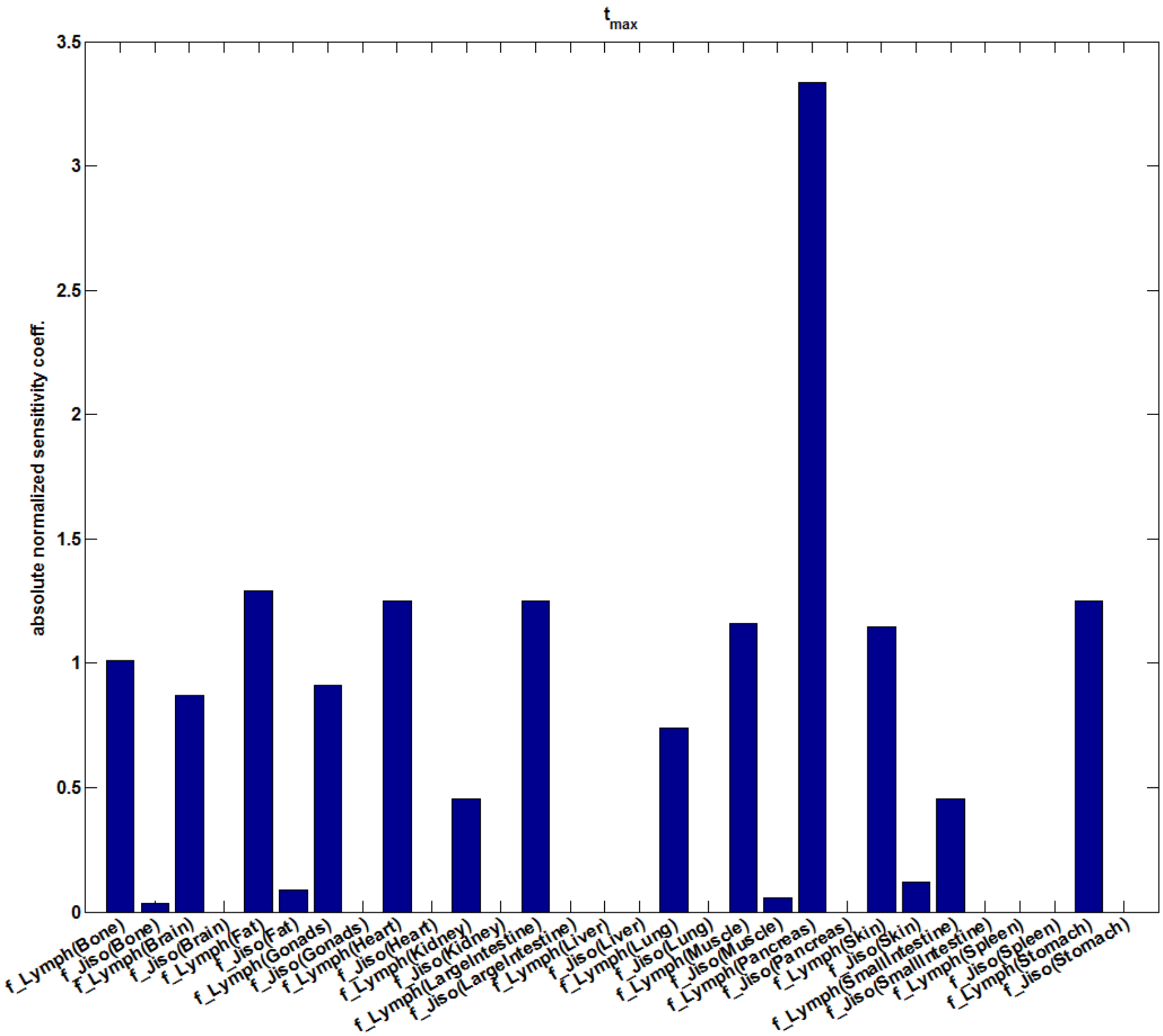

Figure S3: Sensitivity coefficients for the time of maximum concentration of the tissue concentrations of the respective organs for an antibody in mice (7E3 in wild type mice) for lymph (f_Lymph) and fluid recirculation flow (f_Jiso) proportionality factors of the different organs.

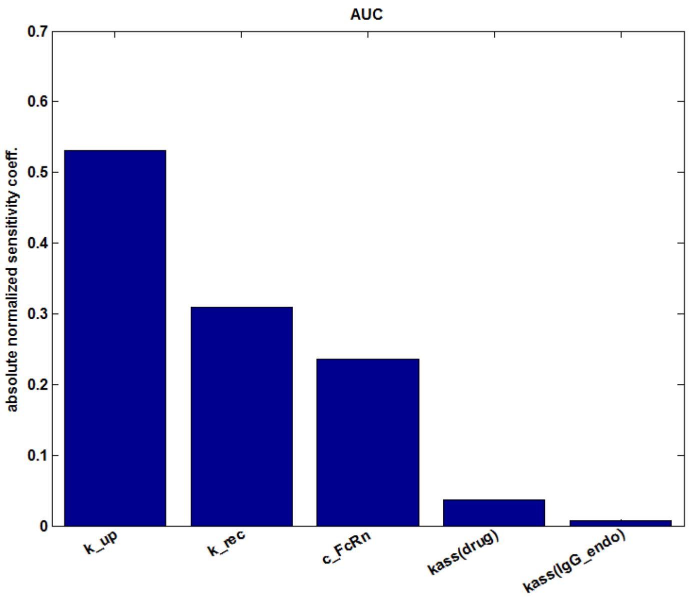

Figure S4: Sensitivity coefficients for the AUC of endosomal clearance / FcRn related parameters (rate constant for endosomal uptake/recycling, FcRn concentration and association rate constants for binding to FcRn) regarding plasma concentration for an antibody in mice (7E3 in wild type mice).

Table S4: 95% confidence intervals (95% CI) and coefficients of variation (CV) of lymph flow proportionality factors f_{lymph} obtained by parameter estimation.

Organ	Parameter value f_{lymph}	95% CI	CV(%)
Bone	6.62E-4	6.62E-4 ± 5.6E-4	43,5
Brain	7.27E-5	7.27E-5 ± 4.9E-5	34,3
Fat	7.54E-3	7.54E-3 ± 3.4E-3	23,2
Gonads	1.11E-2	1.11E-2 ± 4.9E-4	2.3
Heart	1.47E-3	1.47E-3 ± 2.6E-3	90.9
Kidney	7.09E-4	7.09E-4 ± 6.3E-4	45.0
Large Intestine	1.44E-2	1.44E-2 ± 8.0E-3	28.4
Liver	1.99E-2	1.99E-2 ± 8.1E-4	2.1
Lung	3.56E-5	3.56E-5 ± 3.5E-5	50.2
Muscle	2.01E-3	2.01E-3 ± 7.6E-4	19.4
Pancreas	3.03E-2	3.03E-2 ± 2.3E-4	0.4
Skin	3.52E-3	3.52E-3 ± 9.5E-4	13.8
Small Intestine	1.95E-3	1.95E-3 ± 6.6E-4	17.3
Spleen	1.99E-2	1.99E-2 ± 8.1E-4	2.1
Stomach	2.04E-3	2.04E-3 ± 2.8E-3	70.6

Table S5: 95% confidence intervals (95% CI) and coefficients of variation (CV) of fluid recirculation flow proportionality factors f_{Jiso} obtained by parameter estimation.

Organ	Parameter value f_{Jiso}	95% CI	CV (%)
Bone	0.960	0.960 ± 1.6E-5	8,3E-04
Brain	0.404	0.404 ± 2.1E-5	2,7E-03
Fat	0.357	0.357 ± 4.3E-5	6,1E-03
Gonads	0.960	0.960 ± 1.9E-5	9,9E-04
Heart	0.960	0.960 ± 1.4E-5	7,5E-04
Kidney	0.761	0.761 ± 3.6E-5	2,4E-03
Large Intestine	0.179	0.179 ± 4.2E-4	1,2E-01
Liver	0.960	0.960 ± 2.1E-5	1,1E-03
Lung	0.010	0.010 ± 7.1E-7	3,6E-03
Muscle	0.292	0.292 ± 5.0E-5	8,7E-03
Pancreas	0.010	0.010 ± 5.9E-5	3,0E-01
Skin	0.617	0.617 ± 6.6E-5	5,5E-03
Small Intestine	0.179	0.179 ± 4.2E-4	1,2E-01
Spleen	0.010	0.010 ± 8.2E-6	4,2E-02
Stomach	0.960	0.960 ± 1.9E-5	1,0E-03

Table S6: 95% confidence intervals (95% CI) and coefficients of variation (CV) of endosomal clearance / FcRn related parameters obtained by parameter estimation.

	Parameter value	95% CI	CV (%)
Free endosomal FcRn concentration in mice [µmol/L]	38.7	38.7 ± 4.4E-6	5,8E-06
Free endosomal FcRn concentration in monkeys [µmol/L]	21.0	21.0 ± 1.4E-6	3,5E-06
Free endosomal FcRn concentration in humans [µmol/L]	80.8	80.8 ± 1.8E-8	1,1E-08
k_{up} [min-1]	0.294	0.294 ± 6.6E-4	1,1E-01
k_{rec} [min-1]	0.0888	0.0888 ± 1.5E-3	8,8E-01
k_{ass} [L/µmol/min]	0.87	0.87 ± 4.4E-5	2,6E-03

4. Contribution of convection and diffusion to extravasation

The relative contributions from convection and diffusion via large and small pores as calculated by the Peclet number are given for the antibody 7E3 in mice (Table S7), the domain antibody dAb2 in mice (Table S8) and inulin in rats (Table S9).

Table S7: Relative contribution of convection and diffusion to extravasation in different organs for the antibody 7E3 in mice (wild type).

Organ	Relative contribution convection small pores [%]	Relative contribution diffusion small pores [%]	Relative contribution convection large pores [%]	Relative contribution diffusion large pores [%]
Bone	0,0038	6,70E-14	79	21
Brain	0,16	3,20E-13	99	0,55
Fat	0,18	1,70E-14	95	5,2
Gonads	0,0046	1,40E-14	96	4,2
Heart	0,0045	1,20E-14	94	6,1
Kidney	0,024	7,20E-15	69	31
Large Intestine	0,56	5,60E-15	63	36
Small Intestine	0,15	1,60E-14	14	86
Liver	0,12	4,3	57	39
Lung	0,59	3,50E-13	31	68
Muscle	0,19	7,10E-14	78	22
Pancreas	1,2	3,30E-14	63	36
Skin	0,064	1,20E-14	92	7,8
Spleen	3,7	3,9	57	35
Stomach	0,0046	3,20E-15	96	4,4

Table S8: Relative contribution of convection and diffusion to extravasation in different organs for the domain antibody dAb2 in mice.

Organ	Relative contribution convection small pores [%]	Relative contribution diffusion small pores [%]	Relative contribution convection large pores [%]	Relative contribution diffusion large pores [%]
Bone	0,051	92	4	3,7
Brain	22	25	52	1
Fat	7,7	74	15	3
Gonads	0,25	77	20	3,1
Heart	0,18	82	14	3,3
Kidney	0,22	94	2,4	3,7
Large Intestine	4,2	90	1,8	3,6
Small Intestine	0,49	96	0,17	3,8
Liver	0,11	44	18	38
Lung	2,4	93	0,48	3,7
Muscle	2,4	90	3,7	3,6
Pancreas	8,6	86	1,7	3,4
Skin	2,1	83	11	3,3
Spleen	3,5	42	19	36
Stomach	0,24	78	19	3,1

Table S9: Relative contribution of convection and diffusion to extravasation in different organs for inulin in rats.

Organ	Relative contribution convection small pores [%]	Relative contribution diffusion small pores [%]	Relative contribution convection large pores [%]	Relative contribution diffusion large pores [%]
Bone	0,14	99	0,18	0,69
Brain	17	76	7,1	0,53
Fat	2,3	96	0,89	0,67
Gonads	0,8	97	1	0,68
Heart	0,86	97	1,2	0,67
Kidney	0,11	99	0,12	0,69
Large Intestine	2,4	97	0,38	0,67
Small Intestine	0,14	99	0,028	0,69
Liver	0,52	63	4,9	32
Lung	0,37	99	0,031	0,69
Muscle	0,4	99	0,12	0,69
Pancreas	1,8	97	0,15	0,68
Skin	0,88	98	0,53	0,68
Spleen	2,1	59	9,1	30
Stomach	0,78	98	1	0,68

5. Equations for steady state concentrations for FcRn and endogenous IgG

Abbreviations

kep: $f_{vas}^{rec} \cdot k_{rec} \cdot V_{end}$

kpe: $f_{vas}^{up} \cdot k_{up} \cdot V_{end}$

kei: $(1 - f_{vas}^{rec}) \cdot k_{rec} \cdot V_{end}$

kie: $(1 - f_{vas}^{up}) \cdot k_{up} \cdot V_{end}$

kdeg: $(k_{up} - k_{rec}) \cdot V_{end}$

k_conv: $(J_{iso,org} + \alpha_{L,org} L_{org})(1 - \sigma_{L,org}) + (-J_{iso,org} + \alpha_{S,org} L_{org})(1 - \sigma_{S,org})$

k_diff_pls: $PS_{L,org} \cdot \frac{Pe_{L,org}}{e^{Pe_{L,org}-1}} + PS_{S,org} \cdot \frac{Pe_{S,org}}{e^{Pe_{S,org}-1}}$

k_diff_int = k_diff_pls

Cpl: steady state plasma concentration of endogenous IgG

FcRnEnd: steady state endosomal FcRn concentration

ka: k_{ass} (association rate constant for binding to FcRn)

Kdn: K_d(FcRn) for endogenous IgG in neutral environment

Kd: K_d(FcRn) for endogenous IgG in endosomal space

Ql: lymph flow

krpls: $f_{pls}^{mem} \cdot f_{vas}^{up} \cdot k_{up} \cdot V_{end}$

krint: $f_{\text{int}}^{mem} \cdot (1 - f_{vas}^{up}) \cdot k_{up} \cdot V_{end}$

Start concentration of endogenous IgG in interstitial space of the sub-model for endogenous IgG/FcRn

Cpl*(kdeg*kep*k_diff_pls+kdeg*kep*k_conv+ka*FcRnEnd*Ve*kei*k_diff_pls+ka*FcRnEnd*Ve*kei*k_conv+kdeg*kei*k_conv+ka*FcRnEnd*Ve*kep*k_diff_pls+ka*FcRnEnd*Ve*kep*k_conv+kdeg*Kd*ka*Ve*k_diff_pls+kdeg*Kd*ka*Ve*k_conv+kpe*ka*FcRnEnd*Ve*kei+kdeg*kei*k_diff_pls)/(kdeg*kep*Ql+kdeg*kep*kie+kdeg*kep*k_diff_int+ka*FcRnEnd*Ve*kei*Ql+ka*FcRnEnd*Ve*kei*k_diff_int+ka*FcRnEnd*Ve*kep*Ql+ka*FcRnEnd*Ve*kep*kie+ka*FcRnEnd*Ve*kep*k_diff_int+kdeg*Kd*ka*Ve*Ql+kdeg*Kd*ka*Ve*kie+kdeg*Kd*ka*Ve*k_diff_int+kdeg*kei*Ql+kdeg*kei*kie+kdeg*kei*k_diff_int)

Start concentration of endogenous IgG in endosomal space of the sub-model for endogenous IgG/FcRn

Cpl*(k_diff_int*kpe+kie*k_conv+kie*k_diff_pls+kie*kpe+Ql*kpe)*(Kd*ka*Ve+kep+kei)/(kdeg*kep*Ql+kdeg*kep*kie+kdeg*kep*k_diff_int+ka*FcRnEnd*Ve*kei*Ql+ka*FcRnEnd*Ve*kei*k_diff_int+ka*FcRnEnd*Ve*kep*Ql+ka*FcRnEnd*Ve*kep*kie+ka*FcRnEnd*Ve*kep*k_diff_int+kdeg*Kd*ka*Ve*Ql+kdeg*Kd*ka*Ve*kie+kdeg*Kd*ka*Ve*k_diff_int+kdeg*kei*Ql+kdeg*kei*kie+kdeg*kei*k_diff_int)

Start concentration of endogenous IgG – FcRn complex in endosomal space of the sub-model for endogenous IgG/FcRn

Cpl*ka*FcRnEnd*Ve*(k_diff_int*kpe+kie*k_conv+kie*k_diff_pls+kie*kpe+Ql*kpe)/(kdeg*kep*Ql+kdeg*kep*kie+kdeg*kep*k_diff_int+ka*FcRnEnd*Ve*kei*Ql+ka*FcRnEnd*Ve*kei*k_diff_int+ka*FcRnEnd*Ve*kep*Ql+ka*FcRnEnd*Ve*kep*kie+ka*FcRnEnd*Ve*kep*k_diff_int+kdeg*Kd*ka*Ve*Ql+kdeg*Kd*ka*Ve*kie+kdeg*Kd*ka*Ve*k_diff_int+kdeg*kei*Ql+kdeg*kei*kie+kdeg*kei*k_diff_int)

Start concentration of endogenous IgG – FcRn complex in plasma of the sub-model for endogenous IgG/FcRn

kep*Cpl*FcRnEnd*Ve*(k_diff_int*kpe+kie*k_conv+kie*k_diff_pls+kie*kpe+Ql*kpe)*(ka*Cpl*Vpls+krpls)/((kdeg*kep*Ql+kdeg*kep*kie+kdeg*kep*k_diff_int+ka*FcRnEnd*Ve*kei*Ql+ka*FcRnEnd*Ve*kei*k_diff_int+ka*FcRnEnd*Ve*kep*Ql+ka*FcRnEnd*Ve*kep*kie+ka*FcRnEnd*Ve*kep*k_diff_int+kdeg*Kd*ka*Ve*Ql+kdeg*Kd*ka*Ve*kie+kdeg*Kd*ka*Ve*k_diff_int+kdeg*kei*Ql+kdeg*kei*kie+kdeg*kei*k_diff_int)*krpls*Kdn*Vpls)

Start concentration of endogenous IgG – FcRn complex in interstitial space of the sub-model for endogenous IgG/FcRn

kei*Cpl*FcRnEnd*Ve*(k_diff_int*kpe+kie*k_conv+kie*k_diff_pls+kie*kpe+Ql*kpe)*(krint*kdeg*Kd*ka*Ve*k_diff_int+krint*kdeg*kei*Ql+krint*kdeg*Kd*ka*Ve*kie+krint*kdeg*kei*kie+krint*kdeg*Kd*ka*Ve*Ql+krint*kdeg*kei*k_diff_int+krint*ka*FcRnEnd*Ve*kep*k_diff_int+krint*ka*FcRnEnd*Ve*kep*kie+krint*ka*FcRnEnd*Ve*kep*Ql+ka^2*Cpl*Vint*kpe*FcRnEnd*Ve*kei+ka^2*Cpl*Vint*kdeg*Kd*Ve*k_conv+ka^2*Cpl*Vint*kdeg*Kd*Ve*k_diff_pls+ka*Cpl*Vint*kdeg*kei*k_diff_pls+ka^2*Cpl*Vint*FcRnEnd*Ve*kep*k_conv+ka^2*Cpl*Vint*FcRnEnd*Ve*kep*k_diff_pls+ka*Cpl*Vint*kdeg*kei*k_conv+krint*kdeg*kep*Ql+ka^2*Cpl*Vint*FcRnEnd*Ve*kei*k_conv+ka^2*Cpl*Vint*FcRnEnd*Ve*kei*k_diff_pls+ka*Cpl*Vint*kdeg*kep*k_conv+krint*kdeg*kep*kie+ka*Cpl*Vint*kdeg*kep*k_diff_pls+krint*kdeg*kep*k_diff_int+krint*ka*FcRnEnd*Ve*kei*Ql+krint*ka*FcRnEnd*Ve*kei*k_diff_int) / (((kdeg*kep*Ql+kdeg*kep*kie+kdeg*kep*k_diff_int+ka*FcRnEnd*Ve*kei*Ql+ka*FcRnEnd*Ve*kei*k_diff_int+ka*FcRnEnd*Ve*kep*Ql+ka*FcRnEnd*Ve*kep*kie+ka*FcRnEnd*Ve*kep*k_diff_int+kdeg*Kd*ka*Ve*Ql+kdeg*Kd*ka*Ve*kie+kdeg*Kd*ka*Ve*k_diff_int+kdeg*kei*Ql+kdeg*kei*kie+kdeg*kei*k_diff_int)^2*krint*Kdn*Vint))

Start concentration of FcRn in plasma of the sub-model for endogenous IgG/FcRn

kep*Cpl*ka*FcRnEnd*Ve*(k_diff_int*kpe+kie*k_conv+kie*k_diff_pls+kie*kpe+Ql*kpe)/((kdeg*kep*Ql+kdeg*kep*kie+kdeg*kep*k_diff_int+ka*FcRnEnd*Ve*kei*Ql+ka*FcRnEnd*Ve*kei*k_diff_int+ka*FcRnEnd*Ve*kep*Ql+ka*FcRnEnd*Ve*kep*kie+ka*FcRnEnd*Ve*kep*k_diff_int+kdeg*Kd*ka*Ve*Ql+kdeg*Kd*ka*Ve*kie+kdeg*Kd*ka*Ve*k_diff_int+kdeg*kei*Ql+kdeg*kei*kie+kdeg*kei*k_diff_int)*krpls)

Start concentration of FcRn in interstitial space of the sub-model for endogenous IgG/FcRn

kei*Cpl*ka*FcRnEnd*Ve*(k_diff_int*kpe+kie*k_conv+kie*k_diff_pls+kie*kpe+Ql*kpe)/((kdeg*kep*Ql+kdeg*kep*kie+kdeg*kep*k_diff_int+ka*FcRnEnd*Ve*kei*Ql+ka*FcRnEnd*Ve*kei*k_diff_int+ka*FcRnEnd*Ve*kep*Ql+ka*FcRnEnd*Ve*kep*kie+ka*FcRnEnd*Ve*kep*k_diff_int+kdeg*Kd*ka*Ve*Ql+kdeg*Kd*ka*Ve*kie+kdeg*Kd*ka*Ve*k_diff_int+kdeg*kei*Ql+kdeg*kei*kie+kdeg*kei*k_diff_int)*krint)

Production rate of endogenous IgG (IgG_{endo})

Cpl*kdeg*(k_diff_pls*kei*kie+k_diff_pls*Kd*ka*Ve*kie+k_diff_pls*kep*kie+k_conv*kei*kie+k_conv*Kd*ka*Ve*kie+k_conv*kep*kie+kpe*kep*kie+kpe*kep*k_diff_int+kpe*kep*Ql+kpe*Kd*ka*Ve*Ql+kpe*kei*k_diff_int+kpe*kei*Ql+kpe*Kd*ka*Ve*kie+kpe*Kd*ka*Ve*k_diff_int+kpe*kei*kie)/(kdeg*kep*Ql+kdeg*kep*kie+kdeg*kep*k_diff_int+ka*FcRnEnd*Ve*kei*Ql+ka*FcRnEnd*Ve*kei*k_diff_int+ka*FcRnEnd*Ve*kep*Ql+ka*FcRnEnd*Ve*kep*kie+ka*FcRnEnd*Ve*kep*k_diff_int+kdeg*Kd*ka*Ve*Ql+kdeg*Kd*ka*Ve*kie+kdeg*Kd*ka*Ve*k_diff_int+kdeg*kei*Ql+kdeg*kei*kie+kdeg*kei*k_diff_int)

References

1. Klann M, Koeppl H (2012) Spatial simulations in systems biology: from molecules to cells. International journal of molecular sciences 13 (6):7798-7827. doi:10.3390/ijms13067798
2. Sepp A, Berges A, Sanderson A, Meno-Tetang G (2015) Development of a physiologically based pharmacokinetic model for a domain antibody in mice using the two-pore theory. Journal of pharmacokinetics and pharmacodynamics 42 (2):97-109. doi:10.1007/s10928-014-9402-0
3. Venturoli D, Rippe B (2005) Ficoll and dextran vs. globular proteins as probes for testing glomerular permselectivity: effects of molecular size, shape, charge, and deformability. American journal of physiology Renal physiology 288 (4):F605-613. doi:10.1152/ajprenal.00171.2004
4. Taylor AE, Granger DN (1984) Exchange of macromolecules across the microcirculation. Handbook of Physiology The Cardiovascular System Microcirculation 4 (pt 2):467-520
5. Renkin EM (1977) Multiple pathways of capillary permeability. Circ Res 41 (6):735-743
6. Jackson DC, Crabb BS, Poumbourios P, Tulip WR, Laver WG (1991) Three antibody molecules can bind simultaneously to each monomer of the tetramer of influenza virus neuraminidase and the trimer of influenza virus hemagglutinin. Archives of virology 116 (1-4):45-56
7. Malvern Instruments Ltd U (2009) Protein solutions. Hydrodynamic radius determined by dynamic light scattering (DLS). http://www.malvern.com/LabEng/industry/protein/protein_solutions.htm. Accessed 28 August 2009
8. Crone C, Levitt D (1984) Capillary permeability to small solutes. Handbook of physiology 2:411-466
